# Sexual Polyploidization in *Medicago sativa* L.: Impact on the Phenotype, Gene Transcription, and Genome Methylation

**DOI:** 10.1534/g3.115.026021

**Published:** 2016-02-05

**Authors:** Daniele Rosellini, Nicoletta Ferradini, Stefano Allegrucci, Stefano Capomaccio, Elisa Debora Zago, Paola Leonetti, Bachir Balech, Riccardo Aversano, Domenico Carputo, Lara Reale, Fabio Veronesi

**Affiliations:** * Department of Agricultural Food and Environmental Sciences, University of Perugia, Borgo XX giugno 74, 06121, Perugia, Italy; † Department of Biotechnology, University of Verona, Ca' Vignal 1, Strada Le Grazie 15, 37134 Verona, Italy; ‡ Institute for Sustainable Plant Protection, CNR, Via Amendola 122/D, 70126 Bari, Italy; § Istituto di Biomembrane e Bioenergetica, CNR, Via Giovanni Amendola 165/A, 70126 Bari, Italy; ** Department of Agriculture, University of Naples Federico II, via Università 100, 80055 Portici, Naples, Italy

**Keywords:** alfalfa, DNA methylation, polysomic polyploids, tetrasomic inheritance, transcriptome

## Abstract

Polyploidization as the consequence of 2*n* gamete formation is a prominent mechanism in plant evolution. Studying its effects on the genome, and on genome expression, has both basic and applied interest. We crossed two diploid (2*n* = 2x = 16) *Medicago sativa* plants, a *subsp. falcata* seed parent, and a *coerulea × falcata* pollen parent that form a mixture of *n* and 2*n* eggs and pollen, respectively. Such a cross produced full-sib diploid and tetraploid (2*n* = 4x = 32) hybrids, the latter being the result of bilateral sexual polyploidization (BSP). These unique materials allowed us to investigate the effects of BSP, and to separate the effect of intraspecific hybridization from those of polyploidization by comparing 2x with 4x full sib progeny plants. Simple sequence repeat marker segregation demonstrated tetrasomic inheritance for all chromosomes but one, demonstrating that these neotetraploids are true autotetraploids. BSP brought about increased biomass, earlier flowering, higher seed set and weight, and larger leaves with larger cells. Microarray analyses with *M. truncatula* gene chips showed that several hundred genes, related to diverse metabolic functions, changed their expression level as a consequence of polyploidization. In addition, cytosine methylation increased in 2x, but not in 4x, hybrids. Our results indicate that sexual polyploidization induces significant transcriptional novelty, possibly mediated in part by DNA methylation, and phenotypic novelty that could underpin improved adaptation and reproductive success of tetraploid *M. sativa* with respect to its diploid progenitor. These polyploidy-induced changes may have promoted the adoption of tetraploid alfalfa in agriculture.

Polyploidization is an increase in the number of genomes per cell, and occurs in nature as the consequence of 2*n* gamete formation (sexual polyploidization, the most frequent mechanism; Brownfield and Köhler 2010), or as the consequence of somatic genome duplications (somatic polyploidization). A duplication of a species’ chromosomes results in the formation of an autopolyploid having completely homologous duplicated chromosomes. The merging of the genomes of two species, concomitant with genome doubling, results in the formation of an allopolyploid. Polyploidy is widespread in plants, to the point that at least 70% of species have experienced polyploidization at some point of their evolution, and about 50% of economically important species are polyploid (Wendel 2000; Bowers *et al.* 2003; Wood *et al.* 2009).

The effects of polyploidization have been studied widely at the genetic, cytogenetic, and phenotypic level (New Phytologist 2010; Osborn *et al.* 2003; Chen 2007; Doyle *et al.* 2008; Hegarty and Hiscock 2008; Aversano *et al.* 2012; Tayalé and Parisod 2013; Cheng *et al.* 2015). These studies have shown that gene expression changes can be brought about by polyploidization in many ways: gene dosage modification (copy number increase), alteration of the interactions among transcription factors, histone and chromatin state modifications, and DNA cytosine methylation. All these phenomena can translate into silencing or activation of genes and transposable elements, which, in turn, may result in novel traits such as increased cell size, changes in growth habit, or flowering time.

It is reasonable to expect that autopolyploidy, a duplication of existing genes, does not result in modifications as deep as those caused by allopolyploidy, which involves the merging of genomes of different species (Guo *et al.* 1996; Wang *et al.* 2004, 2006; Madlung *et al.* 2005; Parisod *et al.* 2010; Pignatta *et al.* 2010). It is becoming accepted that hybridization, both intraspecific and interspecific, is a more powerful trigger of genomic and gene expression novelties than polyploidization *per se* (Albertin *et al.* 2006; Hegarty *et al.* 2006; Wang *et al.* 2006; Miller *et al.* 2012).

Research on polyploidy has been devoted mainly to allopolyploids, with comparatively little work in autopolyploids. In addition, in the vast majority of published research, polyploids were produced through somatic doubling, whereas, in nature, sexual events involving 2*n* gametes represent the main route to polyploid formation. In light of this, more data on the effect of autopolyploidization would be useful (Stupar *et al.* 2007; Allario *et al.* 2011; Aversano *et al.* 2015).

Alfalfa (*Medicago sativa* L, 2*n* = 4x = 32) is a widely cultivated autotetraploid forage species with tetrasomic inheritance (Quiros 1982; Julier *et al.* 2003). The cultivated form is mostly *Medicago* x *varia*, originated by the hybridization of *M. sativa* subsp. *sativa* with *M. sativa* subsp. *falcata* (Small 2011). These subspecies have distinctive traits: subsp. *sativa* has purple flowers and coiled pods, and is adapted to warm and dry climates, whereas subsp. *falcata* has yellow flowers and sickle-shaped pods, and is adapted to cool and humid environments. The natural distribution of the two subspecies has overlapping areas, including Transcaucasia, Turkey, Iran and Southern Turkistan, where alfalfa is thought to have been initially cultivated about 8000–9000 yr ago. Both subspecies exist at the diploid (2x) and tetraploid (4x) level, but the diploids are not cultivated (reviewed by Small 2011). Sexual polyploidization is thought to be the mechanism by which tetraploid alfalfa originated (Barcaccia *et al.* 2003; Veronesi *et al.* 1986).

The objective of this work was to investigate the consequences of sexual polyploidization in alfalfa. We crossed two previously selected diploid (2x) plants, a *M. sativa subsp. falcata* seed parent, and a *M. sativa coerulea x falcata* pollen parent. Both are spontaneous meiotic mutants, producing a mixture of *n* and 2*n* eggs and pollens, respectively. Such a cross produced full-sib 2x and 4x hybrids, the latter being the result of bilateral sexual polyploidization (BSP). These unique materials allow us to investigate the effects of BSP, and separate the effects of intraspecific hybridization from those of polyploidization by comparing 2x *vs.* 4x full sibs. To make sure that they were true autopolyploids, we first characterized chromosome pairing behavior (random *vs.* preferential pairing) of the neopolyploids, by assessing segregation of simple sequence repeat (SSR) markers. Then, polyploidization-induced changes in leaf and leaf cell morphology, biomass production, and fertility traits were described. Finally, gene expression and epigenetic changes were studied from a genome-wide perspective by microarray and methylation-sensitive amplified polymorphism (MSAP) markers, respectively. Our findings contribute to understanding the success of polyploid *M. sativa* in agriculture, and could have practical implications in breeding of alfalfa and other polyploids.

## Materials and Methods

### Plant material and ploidy determination

**Figure 1:**
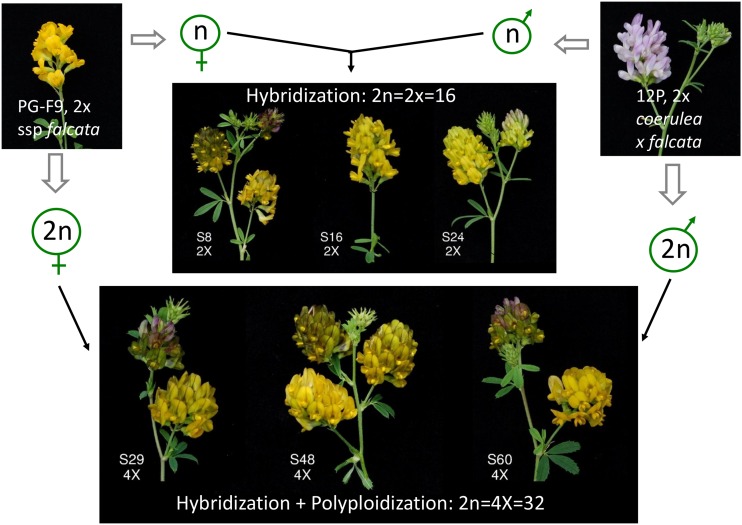
Obtaining diploid and tetraploid hybrids. PG-F9 and 12P are diploid plants that produce a significant percentage of 2*n* eggs and pollen, respectively. The PG-F9 × 12P cross produced 2x and 4x hybrids. The typical greenish flower color derives from crossing yellow-flowered PG-F9 with purple-flowered 12P.

Two *M. sativa* meiotic mutants made this study possible. The *M. sativa* subsp. *falcata* genotype PG-F9 produces 55–70% 2*n* eggs (Tavoletti 1994; Barcaccia *et al.* 1997). The *M. sativa* genotype 12P was obtained by two cycles of recurrent selection for 2*n* pollen production from a cross of *M. sativa* subsp. *coerulea* and *M. sativa* subsp. *falcata* (Tavoletti *et al.* 1991). PG-F9 and 12-P were cloned by cuttings, reared in pots in a greenhouse under natural light, and crossed without emasculation using PG-F9 as the female parent. At maturity, seeds were harvested, and 200 of them were sown in jiffy pots. The plants were reared in flats in a greenhouse at the Department of Agricultural, Food and Environmental Sciences, University of Perugia under continuous illumination. Among the PG-F9 × 12-P hybrids, about 5% 4x and very few 3x hybrids are expected (Barcaccia *et al.* 1998). For the screening of ploidy level, a quick test based on chloroplast counts of guard cells was employed (Bingham 1968) on 10 plants displaying a “2x phenotype” (small, narrow leaflets), and 10 displaying a “4x phenotype” (large, wide leaflets). To confirm chromosome number, root tips were used to count mitotic chromosomes of six putative 2x and 4x plants as previously described (Barcaccia *et al.* 1995). Three randomly taken plants per ploidy level were used in this study. Greenish flower color of these plants (Figure 1) confirmed that they derived from crossing, and not from selfing of the PG-F9 female parent.

### Chromosome pairing behavior of BSP neopolyploids

The three 4x BSP plants (S29, S48, S60) were crossed with a pollen donor from the Italian variety Classe (4x). Sixty plants per progeny were reared in a greenhouse until genomic DNA was extracted (SIGMA Genelute plant kit). Twenty eight published SSR markers (Diwan *et al.* 2000, Julier *et al.* 2003; Sledge *et al.* 2005; Mun *et al.* 2006) were tested for their ability to provide parent-specific alleles, that is, alleles present in only one of the diploid parents and not shared with the tetraploid tester (Classe). Primers were selected based on chromosome location. Amplifications were performed as follows: buffer 1X, MgCl_2_ 1.5 mM, dNTP 0.2 mM, primer FOR/REV 0.5 µM, *Taq* polymerase (Sigma) 1 U, genomic DNA 30 ng, in 20 µl final volume. PCR cycling was 94° 3 min, 40 cycles at 94° 30 sec, Ta °C 30 sec, 72° 30 sec, where Ta is the marker-specific annealing temperature (Supplemental Material, Table S1). After screening in agarose, fluorescein isothiocyanate (FITC)-labeled primers for nine selected primer pairs (Table S1) were used for amplification of the three parental plants and the three hybrids (60 plants each), and capillary electrophoresis was performed (3130x Genetic Analyzer, Applied Biosystems). Electrophoretic data were analyzed using the software Gene Mapper 4 (Applied Biosystems). The expected segregations of the markers were determined, under the assumption of no double reduction, for the hypotheses of complete preferential pairing, or complete random pairing (Figure S1), and χ^2^ values for goodness of fit were calculated.

### Leaf morphology, biomass and fertility assessment

The parents and the selected 2x and 4x progeny plants were cloned from cuttings. Eight rooted cuttings per genotype were reared in pots containing a soil:sand:neutral peat moss (3:1:1) mix in the greenhouse with natural light during late winter to early spring with complete randomization. Fresh and dry matter yield (g per plant) was assessed after clipping the plants, when about 10 stems had open flowers; due to differences in flowering times among genotypes, harvesting was not performed at the same time. Dry matter yield was determined after desiccating the fresh material for 48 hr at 100°. Biomass yield was evaluated again in two subsequent regrowth cycles in a screen house with drip irrigation during the summer under natural sunlight. Flowering time was evaluated as days from March 1 (spring assessment), from June 1 (first summer assessment), and as days from the previous cut (second summer assessment). Pollen production per floret, estimated visually by tripping 10 random florets per plant was scored from 0 (no visible pollen), to 3 (abundant pollen). Pollen fertility and diameter were assessed by mixing pollen from four random florets per genotype, staining it on a microscope slide with acetocarmine. Digital pictures were taken using a Leica DMLP optical microscope equipped with a Leica ICCA digital camera. The percentage of stained grains was calculated on three random microscope fields. Pollen diameter was measured using the Leica IM1000 software. Seed set was assessed in the greenhouse during the winter and spring using continuous illumination (sodium halide lamps) and, on the regrowth of the same plants, during the summer in a screen house with natural light. Ovule fertility was estimated by assessing callose accumulation within the nucellus in ovules at flower maturity, as described by Rosellini *et al.* (1998). Four racemes (replicates) per plant were crossed without emasculation using three unrelated *M. sativa* subsp. *coerulea* (2x), and one unrelated cultivated *M. sativa* subsp. *sativa* (variety Classe) pollen donors were used for crosses. All plants were fertilized with pollen from both the 2x and the 4x pollen donors, thus performing intraploidy (2x-2x, 4x-4x), and interploidy (2x-4x, 4x-2x), crosses. Hand-crosses in all combinations were also made between 2x hybrids, and between 4x hybrids (full sib crosses). Self-fertility was estimated by hand-tripping florets of two to four racemes per plant. Seed set was estimated by calculating the number of seeds per floret.

Digital pictures of leaves, flowers, and mature pods were taken for each plant. Leaf shape was assessed as the ratio of width to length of the central leaflet of five random fully expanded leaves per genotype. Pieces of leaf lower epidermis were peeled off using fine forceps, and placed onto a microscope slide in a drop of water. Digital pictures of the epidermal cells were taken, and the surface area of cells surrounding the stomata measured with the equipment described above. The length and width of the stomata guard cells were also measured, and stomata surface area calculated.

All the morphological, biomass and fertility data were subjected to ANOVA using the GLM procedure of SAS (Statistical Analysis System, Inc. Cary, NC, 2009), using a fixed model in which “plant group” was the classification variable, with three levels: parents, 2x, and 4x hybrids, respectively. Means were separated using LSMeans with the PDIFF option, with a significance threshold of *P* = 0.05.

### Microarray analyses

Six cloned plants of PG-F9, 12P, and their 2x and 4x hybrids, were reared in a screen house under natural conditions, with complete randomization. Young, fully expanded leaves were harvested from shoots of each plant at the vegetative stage, bulking the leaves of two plants, thus obtaining three biological replicates per genotype. The samples were immediately frozen in liquid nitrogen and stored at –80°. About three trifoliate leaves per sample were finely ground in liquid nitrogen, and total RNA was further purified with the Qiagen RNeasy minikit. RNA was quantified on a NanoDrop ND-1000 spectrophotometer, and quality checked using a 2100 Bioanalyzer (Agilent Technologies). Twenty four RNA samples were used for microarray hybridizations. Nimblegen Microarrays were designed at the Centro di Genomica Funzionale, University of Verona, Italy. Probes for 41575 *M. truncatula* genes (TC or singleton ET) were designed based on the *Medicago* Gene Index, release 11.0). For 41,340 genes, it was possible to design three different probes per gene, whereas for the remaining 184 genes, one or two probes were designed.

Labeling and hybridization was performed according to Nimblegen gene expression user guide version 3.2. Scanning was performed with Axon GenePix 4400A scanner. Scanner settings were set according to Nimblegen gene expression user guide version 3.2. Raw data were quantile normalized and summarized with the RMA algorithm (Irizarry *et al.* 2003), as implemented in Nimblescan 2.5 software using default parameters (Nimblegen).

Differential expression analysis was performed using Linear Models Microarray Analysis (LIMMA; Smyth 2005) as follows. To identify genes affected by hybridization, two groups of samples corresponding to parents (PG-F9, three replicates; 12P, three replicates), and to 2x hybrids (s8-2x, three replicates; s16-2x, three replicates; s24-2x, three replicates) were contrasted using a two-class unpaired design. To identify genes affected by hybridization and polyploidization, two groups of samples corresponding to parents (as above), and to 4x hybrids (s29-4x, three replicates; s48-4x, three replicates; s60-4x, three replicates) were contrasted using a two-class unpaired design. To identify genes affected by polyploidization, two groups including 2x hybrids (as above) and 4x hybrids (as above) were contrasted using a two-class unpaired design. Normalized expressions, and SDs for probe sets that were differentially expressed between parental and hybrid progeny means, and fold change and significance (false discovery rate), of each paired comparison are presented in Table S17.

To estimate genotype-specific transcriptional differences, the following pairwise contrasts were performed using a two-class unpaired design: PG-F9 *vs.* 12P; S8-2x *vs.* S16-2x, S8-2x *vs.* S24-2x, S16-2x *vs.* S24-2x; S29-4x *vs.* S48-4x, S29-4x *vs.* S60-4x, and S48-4x *vs.* S60-4x. Genes were considered as differentially expressed among the groups compared if |log2FC|>1 and adjusted *P*-value < 0.05 (Benjamini and Hochberg 1995).

The Blast2GO software (Conesa and Götz 2008; https://www.blast2go.com/) was used to perform a semi-automatic Gene Ontology (GO) annotation, and data mining of probe sequence sets of the PS genes, in order to enhance knowledge on the unannotated sequences. The Biological Networks Gene Ontology tool (BiNGO, Maere *et al.* 2005)—a plugin for Cytoscape (Shannon *et al.* 2003)—was adopted to identify enrichment of GO terms in PS genes with respect to all genes in the microarray. The BinGO analysis tests the probability that the frequency of a GO term in a set of genes (test set) taken from a larger set of genes (reference set) is different from the frequency in the reference set.

### Quantitative (q)RT-PCR analyses

To confirm the results of microarray analyses, six genes from those evidenced by the Bingo analysis were tested by qRT-PCR performed in a Mx3000P Stratagene system (Agilent Technologies Inc., Santa Clara, CA) using FastStart SYBR Green Master Mix (Roche Life Science, Italy) with the following settings: 2 min at 95°, followed by 40 cycles at 95° for 30 s, 58° for 1 min, and 72° for 20 s. For each genotype, three reactions were run from a cDNA synthesis, and the mean values calculated. Primers were designed using the Primer3Plus Software (Table S15). The specificity of amplicons (Table S16) was confirmed by dissociation curve analysis, generated after the last PCR cycle, and by sequencing. Data analysis and calculations, to compare transcript accumulation data, were performed through the 2-ΔΔCt (threshold cycle) method, with β-actin as the endogenous reference.

### MSAP analyses

DNA from leaves of plants used for microarray analysis was purified using the DNeasy Plant Maxi Kit (Qiagen, Valencia, CA) according to the manufacturer’s instructions. DNA purity was evaluated using a NanoDrop ND-1000 spectrophotometer (Thermo Scientific, Wilmington, DE), and nucleic acids concentrations were determined using a Qubit fluorometer (Life Technologies, Carlsbad, CA). The methylation pattern at 5′-CCGG sites was analyzed using the MSAP technique, which is based on the use of the isoschizomeric restriction enzymes *Hpa*II and *Msp*I, which recognize the same restriction site (5′-CCGG-3′), but have different sensitivities to methylation of the cytosine residues. In particular, *Hpa*II digests only nonmethylated CCGG sequences and hemi- (single strand) methylated mCCGG sequences of all possible methylated CCGG variants. *Msp*I can cleave nonmethylated CCGG sequences, and hemi- or fully (both strands) methylated CmCGG sequences, but not hemi- and fully methylated mCCGG and mCmCGG sequences (Reyna-López *et al.* 1997; Mann and Smith 1977). MSAP analysis was performed as reported previously (Aversano *et al.* 2012). For selective amplifications, one FAM-labeled *Eco*RI primer (*Eco*RI-TCCA) was combined with six *Hpa*II-*Msp*I primers (*Hpa*II-*Msp*I-AAC, *Hpa*II-*Msp*I-ACA, *Hpa*II-*Msp*I-ACT, *Hpa*II-*Msp*I-AGA, *Hpa*II-*Msp*I-AGC, *Hpa*II-*Msp*I-AGG), for a total of six primer combinations. To have reproducible and clear banding patterns, each amplification was repeated at least three times, and only bands showing consistent amplification were considered. For each position in the gel, the following *Hpa*II-*Msp*I fragment pattern variants, referring to fragment presence (1) or absence (0), were observed: a 1-1 pattern (a fragment of definite length visualized in both the *Hpa*II and *Msp*I lanes) was attributed to digestion by both enzymes at a nonmethylated CCGG site, and, therefore, associated to unmethylated sites; a 1-0 pattern, representing a fragment of definite length visualized in the *Hpa*II, but not in the *Msp*I, lane may be interpreted as two different situations: 1) the cutting of hemi-methylated mCCGG sites with *Hpa*II but not *Msp*I, and 2) the presence of internal hemi-methylated CmCGG site(s) between the cleaved distal CCGG and the *Eco*RI site; in both cases the 1-0 pattern denotes hemi-methylated sites. A 0-1 pattern corresponds to digestion with *Msp*I but not *Hpa*II, and refers to the presence of a fully methylated CmCGG site. Finally, a (0-0) pattern could be caused either by restriction target absence due to a mutated site when genetically distinct samples are compared, or inhibition of digestion with both enzymes at a fully methylated mCmCGG site when another sample shows the presence of a fragment at that position (Schulz *et al.* 2013; Fulneček and Kovařík 2014). Therefore, the 0-0 profile is not informative and was excluded from the analysis to avoid the noise produced by confounding the effects of mutation and methylation. To decipher hyper- and hypo-methylation changes, we adopted the MSAP scoring method of Fulneček and Kovařík (2014). One-way ANOVA was applied to analyze the cytosine methylation level differences in the diploid and tetraploid hybrids using XLSTAT-PRO 7.5.3 software (Addinsoft, http://www.xlstat.com). The Duncan test was performed to compare mean values. To compare *de novo* methylation and demethylation frequencies, the methylation ratio (MR) was calculated as the percent ratio of the number of markers revealing *de novo* methylation over the number of markers revealing demethylation.

### Data availability

The *M. sativa* meiotic mutants, and their 2x and 4x hybrids, are available upon request for research purposes. The *Supplemental Material* files contain 17 tables and 11 figures with detailed information on SSR markers, chromosome segregation data, phenotypic traits, the results of GO, and homology searches of nonadditively expressed genes, and RT-PCR data. Raw phenotypic data are available on request. Gene expression data are available at GEO under the accession number: ID:GSE71559, and are available at this link: http://www.ncbi.nlm.nih.gov/geo/query/acc.cgi?token=gxkdcaaabvgltsx&acc=GSE71559.

## Results

### Chromosome segregation in neotetraploids

Informative SSR markers were found for all eight chromosomes of the three BSP plants, with the exception of chromosome VII in S60-4x (Table S2). In particular, in S29-4x, tetrasomic inheritance was demonstrated for six of the eight chromosomes (Table 1 and Table S3), while segregation of markers of chromosomes II and V fit with both disomic and tetrasomic transmission. In S48-4x (Table 1 and Table S4), chromosomes V and VII showed disomic inheritance. Chromosome VIII showed an intermediate behavior. In S60-4x, chromosome IV deviated significantly from the expectations of the tetrasomic model (Table 1 and Table S5) due to segregation distortion rather than to preferential pairing. Overall, in these three BSP plants, preferential pairing appears to be limited, leading to disomic or intermediate inheritance for one to three of the eight chromosomes. Chromosome V showed disomic inheritance, as it consistently tended to pair preferentially in these three BSP plants.

**Table 1:** Summary of the results of χ^2^ analysis of segregation mode of SSR markers mapping on all chromosomes in the three BSP plants

	Significance of Chi Square Values*a*					
Chromosome	S29		S48		S60	
	Disomic	Tetrasomic	Disomic	Tetrasomic	Disomic	Tetrasomic
I	**	NS	**	NS	**	NS
II	*	**	NT	NS	**	NS
III	**	NS	**	NS	NT	NS
IV	NT	NS	NT	NS	NT	**
V*b*	*	*	NS	**	NS	**
	**	NS	NS	**		
VI	**	NS	NT	NS	**	NS
VII	**	NS	NS	**	—	—
VIII	**	NS	NS	NS	NT	NS

* Significant at *P* ≤ 0.05; **significant at *P* ≤ 0.01. NS, not significant; NT, nontestable (because one or more of the expected numbers is 0).

a See Table S3, Table S4, and Table S5 for marker segregation data in single progenies of the three BSP plants.

b Data from two SSR loci of chromosome V are available for plants S29 and S60.

The number of alleles found in 4x BSP plants was less than the sum of the alleles of the parental plants for 17 out of 27 SSR loci (Table S6). Although this may be explained by segregation in parental plants (restitutional meiotic divisions in both parents allow for allele segregation), DNA loss in neopolyploids could also be hypothesized (Song *et al.* 1995; Feldman *et al.* 1997; Shaked *et al.* 2001). In particular, the presence in S29 of only one allele of marker MTIC48 of chromosome V (Table S2), may be explained by loss of the PG-F9 allele.

### Phenotypic effect of hybridization and polyploidization

The effects of hybridization *per se* was estimated by comparing 2x hybrids with the parental mean. Several traits exhibited clear midparent heterosis: leaf area and stomata size were larger, and biomass production was moderately higher in 2x hybrids (Figure 3, A, D, E, H, and J, Table S7, Table S8, Table S9, Table S10, Table S11, and Table S12). Stomata density decreased in 2x hybrids (Figure 3, F and G). As for the reproductive traits, 2x hybrids flowered later, had fewer ovules per ovary, and higher seed set than their parents (Figure 4, A, C, and F, and Table S12).

**Figure 2:**
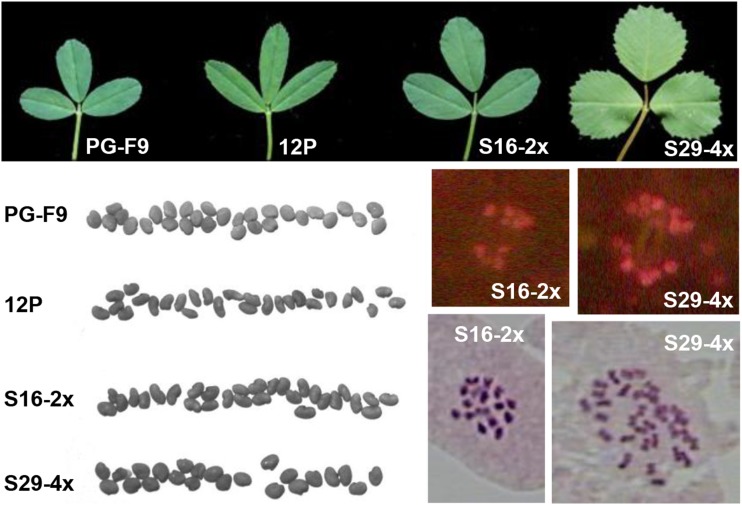
Determination of the ploidy state of 2x and 4x progeny plants (one plant per ploidy state is shown as an example). In 4x plants, leaf size is larger, and leaflets shape are less elongated, seeds are bigger, and the number of chloroplasts in stomata guard cells is higher (for example, S16-2x, 8 chloroplasts; S29-4x, 13 chloroplasts) than in 2x plants. Root tip cell chromosomes counts (for example, S16-2x, 16 chromosomes; S29-4x, 32 chromosomes) confirmed the ploidy estimation based on morphological traits (see text for details).

**Figure 3:**
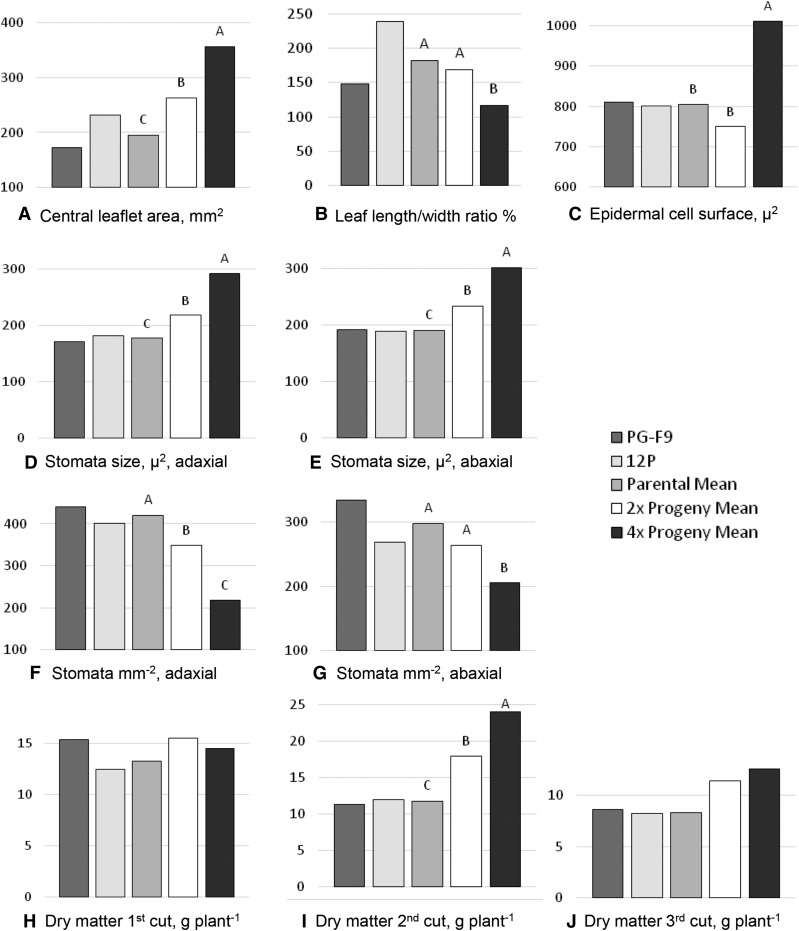
Leaf morphology and dry matter yield of parental genotypes, their mean, and comparisons of the mean with those of 2x and 4x hybrids (three genotypes per ploidy level). (A) Central leaflet surface area (*n* = 20 per genotype). (B) Ratio between length and width of the central leaflet (*n* = 20 per genotype). (C) Surface area of epidermal cells (*n* = 20 per genotype). (D) and (E) Size of stomata of adaxial and abaxial leaf epidermis, calculated from their axes length. (F) and (G) Number of stomata per mm^2^ of adaxial and abaxial leaf epidermis (*n* = 20 per genotype), (H)–(J) Dry matter per plant at each of three consecutive cuts at 10% flowering (*n* = 2–6 per genotype). Different letters above columns indicate significant differences at *P* < 0.05 (see text for details on statistical analyses).

**Figure 4:**
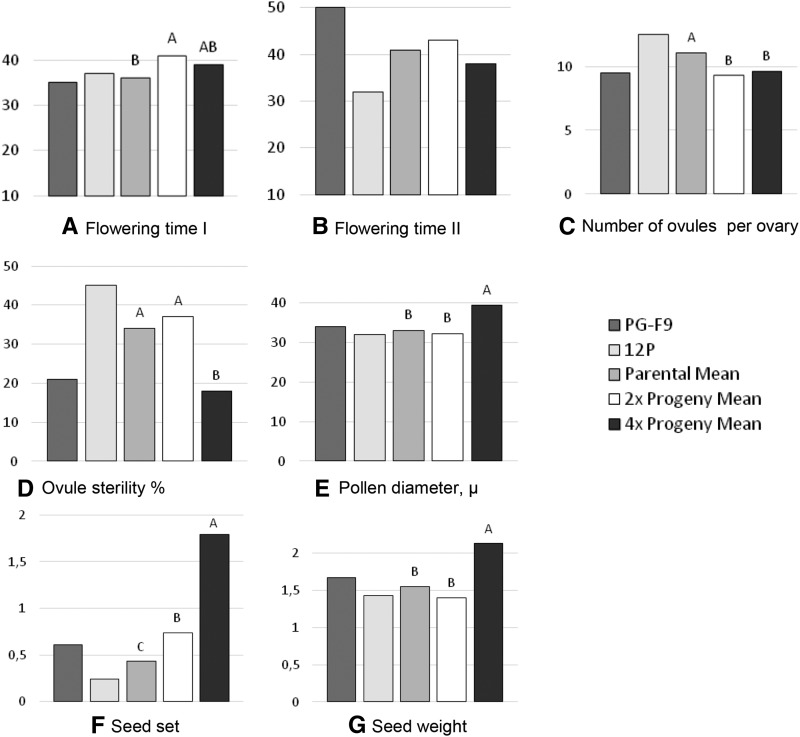
Reproductive traits of parental genotypes, their means, and comparisons with 2x and 4x hybrids (three genotypes per progeny per ploidy level). (A) and (B) Flowering dates of two consecutive regrowths (A, days from June 1; B days from previous cut; *n* = 2–7 per genotype). (C) Number of ovules per ovary (*n* = 20–22 per genotype). (D) Percentage of callosized ovules, estimating ovule sterility (*n* = 20 per genotype). (E) Pollen diameter (*n* = 30–55 per genotype). (F) Seeds produced per floret upon hand pollinations with an unrelated pollen parent of the same ploidy level (*n* = 8–30 florets for each of four racemes, used as replications). Genotype by environment and ploidy × environment interactions for seed set were not significant (not shown), so the mean data across environments are presented (Table S12). (G) Weight of 1000 seeds (*n* = 23–291 per genotype). Different letters above columns indicate significant differences at *P* < 0.05 (see text for details on statistical analyses).

We estimated the added effect of polyploidization to hybridization by comparing full-sib 4x with 2x hybrids. Leaf size, epidermal cell surface and stomata size were increased by tetraploidization (Figure 3, A, and C–E), and stomata density was concomitantly strongly reduced (Figure 3, D–E and Table S7). Leaflet length/width ratio differed between the 4x and the 2x hybrids due to a less elongated shape (Figure 2 and Figure 3C). Green and dry biomass of 4x hybrids was higher than that of 2x hybrids (second cut, Figure 3, H–J, Table S8, Table S9, and Table S10). Delayed flowering observed in 2x hybrids was not present in 4x hybrids. Flowers and pollen grains of 4x hybrids were larger, and ovule sterility lower (Figure 1, Figure 4, D and E, and Table S12). Surprisingly, in intraploidy crosses, seed set of 4x hybrids (1.97 seeds per floret) was 2.7-fold higher than that of 2x hybrids, and seeds were 37% heavier (Figure 2 and Figure 4G). In particular, 4x plants S48 and S60 produced more seeds per floret than both parents (Table S12), consistent with a “high-parent polyploid heterosis” behavior. When the hybrids were pair-crossed in all combinations within ploidy level (full-sib crosses, Table S10), the picture was reversed: 4x hybrids had much lower seed set than 2x hybrids (Table S12).

### Effect of hybridization and polyploidization on gene transcription

**Figure 5:**
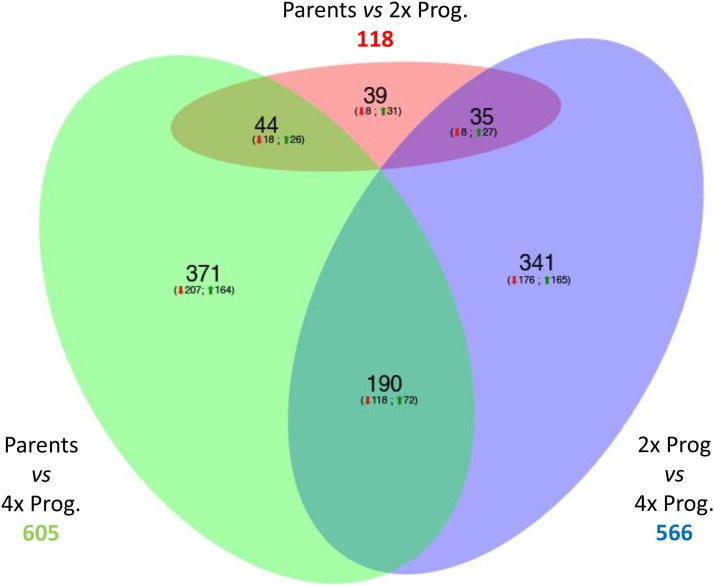
Venn diagram summarizing the results of the microarray experiment. Pink: genes differing in transcription level between the parents and the 2x progeny (Hybridization-sensitive, HS). Green: genes differing between the parents and the 4x hybrids (Hybridization and Polyploidization sensitive, HPS). Blue: genes differing between the 2x and the 4x hybrids (Polyploidization sensitive, PS). The red arrows refer to subset of genes that are underexpressed in the former group of each comparison, while the green arrows refer to the subset of genes that are overexpressed in the former group of each comparison.

In 2x hybrids, nonadditive gene transcription was recorded for only 118 genes (Figure 5). We defined them as “hybridization-sensitive” (HS), because their expression deviated from the parental mean as a consequence of hybridization. When the parents were compared with their 4x hybrids, 605 genes were nonadditively expressed; since their expression was affected by the combined effects of hybridization and polyploidization, we defined them as “hybridization- and polyploidization-sensitive” (HPS). Comparison of gene expression of 2x and 4x hybrids revealed that 566 genes were differentially expressed, 341 of which only between 2x and 4x hybrids, and not between parents and 4x hybrids. Therefore, they are candidates for being affected only by the ploidy change, and not by concurrent hybridization. We defined them as “polyploidization-sensitive” (PS), and will examine them in more detail below.

**Figure 6:**
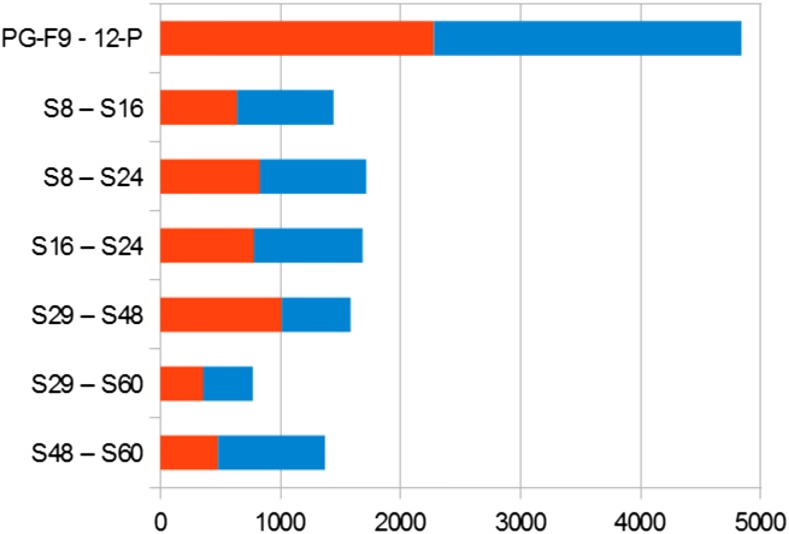
Number of differentially expressed genes in single genotype comparisons. The red bar corresponds to the genes overexpressed in the first genotype of each comparison, the blue bar to the genes overexpressed in the second genotype of each comparison.

The small number of HS genes is probably due to the large transcriptional difference between the parents (Figure 6): for many genes, the parental mean was affected by a large SE, and this restricted the number of statistically significant instances in parent–progeny comparisons. However, the number of HPS genes was about five times that of HS genes, clearly indicating that polyploidization, combined with hybridization, has a significant effect on gene expression.

The comparison of single progeny genotypes with the parental mean (Table 2) showed that there were more overexpressed than underexpressed genes in all genotypes but one (S60-4x). The ratio between the numbers of over and underexpressed genes was 2.13 for 2x and 1.20 for 4x hybrids, indicating that hybridization mostly increased gene transcription, but hybridization combined with polyploidization resulted in a modest increase of the transcription level.

**Table 2:** Numbers of genes whose transcription levels differed significantly between single progeny plants and the parental mean

	2x Hybrids				4x Hybrids			
	S8-2x	S16-2x	S24-2x	Mean	S29-4x	S48-4x	S60-4x	Mean
Overexpressed (O)	236	240	562	346	432	818	393	547
Underexpressed (U)	74	117	490	227	299	680	404	461
Ratio O/U	3.19	2.05	1.15	2.13	1.44	1.20	0.97	1.20
Differentially expressed (O+U)	310	357	1052	573	731	1498	797	1009
Percentage differentially expressed	0.75	0.86	2.53	1.38	1.76	3.61	1.92	2.43

Due to large genetic distance, wide transcriptional differences between the parental genotypes were expected; in fact, 4839/41,538 = 11.6% of the genes differed significantly (Figure 6 and Table S13). The differences between single genotypes within the 2x and 4x hybrids were comparatively small, involving 357–1010 genes. This is consistent with the high genetic similarity between the full-sib plants of each progeny.

### Gene ontology analysis and term enrichment of PS genes

Of 341 PS genes, 240 were annotated (Figure S2). The vast majority of Blast hits were *M. truncatula* sequences, followed by *Glycine max*, *Cicer arietinum* and *Vitis vinifera* (Figure S3). Blast2GO analysis showed that in the “biological process” vocabulary, the more frequent GO term was “oxidation–reduction process” (Figure S4). In the “cellular component” vocabulary, “protein complex” and “integral to membrane” were the most represented terms (Figure S5). In the “molecular function” vocabulary, the most frequent term was “ATP binding”, and related terms were also present (“nucleoside-triphosphatase activity”, “hydrolase activity, acting on ester bonds”). The term “oxidoreductase” was well represented, and “tetrapyrrole binding” was present (see below) (Figure S6). The GOslim procedure was also adopted to restrict the ontology search to the plant kingdom. In the “biological process” vocabulary, “response to stress” and “biosynthetic processes” were the most frequent terms, followed by “cellular protein modification process” and “signal transduction” (Figure S7). In the “cellular component” vocabulary, the plastid compartment clearly predominated, with the “plastid” and “tylacoid” terms (Figure S8). In the “molecular function” vocabulary, nucleic acid binding was the most frequent function with several terms related to it. “Hydrolase activity” and “protein binding” were also frequent (Figure S9).

We used BinGO to determine whether a GO term was more or less frequent than expected in PS genes with respect to the whole microarray. Six “Molecular function”, 18 “Biological process”, and 16 “Cellular component” GO terms were significantly enriched in the PS genes (Table S14). These 40 terms originate from 25 genes (Table 3); 15 of them had higher expression in 4x than in 2x hybrids, and showed homology with chlorophyll-binding proteins (six genes), lipoxygenases (three genes), heat shock proteins (two genes), ribulose 1,5-bisphosphate carboxylase small subunit, UDP-glucosyltransferase, and photosystem I subunit *Psa*D (one gene each). Therefore, most of the genes in this group are related to photosynthesis. Ten genes were less expressed in 4x than in 2x hybrids, and showed homology with a diverse set of proteins: a protein kinase, a chaperonin, an alpha-dioxygenase, a peroxidase precursor, a glutathione S-transferase, a Myb transcription factor, a replication licensing factor, a disease resistance response protein, GDSL esterase/lipase, and thaumatin-like protein 1a (one gene each) (Table 3). No general pattern of variation with respect to the parents was observed in this sample of genes, and stochastic variation within the progeny group was observed (not shown).

**Table 3:** Transcripts differentially expressed between 2x and 4x hybrids whose GO IDs are significantly enriched in the polyploidization-sensitive group of genes with respect to the genes present on the microarray, according to BiNGO analysis

Gene	Log_2_ 2x/4x Expression	Best Blast Hit*a*	E Value
4x > 2x			
TC172620	−4.419	XP_003622001.1: Heat shock protein Hsp70, *Medicago truncatula*	0.0
TC198142	−2.17	XP_003636970.1: Ribulose bisphosphate carboxylase small chain 3A, *M. truncatula*	3e-118
TC183902	−1.723	AAC25775.1: Chlorophyll *a*/*b* binding protein, *M. sativa*	1e-90
TC177786	−1.697	AAC25775.1: Chlorophyll *a*/*b* binding protein, *M. sativa*	2e-167
TC201583	−1.59	XP_003627181.1: Lipoxygenase, *M. truncatula*	0.0
TC173777	−1.571	XP_003627187.1: Lipoxygenase, *M. truncatula*	0.0
TC195187	−1.537	AAC25775.1: Chlorophyll *a*/*b* binding protein, *M. sativa*	2e-167
TC178665	−1.305	XP_004505250.1: UDP-glucose flavonoid 3-O-glucosyltransferase 7-like, *Cicer arietinum*	0.0
TC174046	−1.293	XP_003610706.1: Photosystem I reaction center subunit II, *M. truncatula*	9e-138
TC178639	−1.226	XP_003627185.1: Lipoxygenase, *M. truncatula*	0.0
TC180095	−1.168	XP_003618482.1: Chlorophyll *a*/*b* binding protein, *M. truncatula*	4e-131
TC188202	−1.158	AAC25775.1: Chlorophyll *a*/*b* binding protein, *M. sativa*	7e-101
TC182218	−1.154	XP_003592196.1: Heat shock protein, *M. truncatula*	1e-180
TC179482	−1.058	XP_003618482.1: Chlorophyll *a*/*b* binding protein, *M. truncatula*	2e-174
TC172508	−1.001	XP_003627196.1: Lipoxygenase, *M. truncatula*	0.0
4x < 2x			
TC179350	1.042	XP_003617150.1: Protein kinase 2B, *M. truncatula*	0.0
TC176016	1.056	XP_003626805.1: 10 kDa chaperonin, *M. truncatula*	0.0
TC185299	1.063	CAH05011.1: Alpha-dioxygenase, *Pisum sativum*	0.0
TC176166	1.078	XP_003594497.1: Peroxidase, *M. truncatula*	0.0
TC200070	1.187	XP_003623202.1: Glutathione S-transferase GST 8, *M. truncatula*	2e-159
TC186568	1.203	XP_003591357.1: Myb-like transcription factor, *M. truncatula*	2e-179
TC183565	1.286	XP_003604514.1: Disease resistance response protein, *M. truncatula*	1e-135
TC179158	1.602	XP_003618998.1: GDSL esterase/lipase, *M. truncatula*	0.0
TC173466	1.983	XP_003609791.1: DNA replication licensing factor mcm2, *M. truncatula*	0.0
TC175139	2.088	XP_003625706.1: Thaumatin-like protein, *M. truncatula*	2e-180

The genes are listed according to decreasing Log_2_ 2x/4x expression ratio: negative values mean that the gene has higher expression in 4x that in 2x plants, and vice-versa. The best blast hit of each transcript and its E value are presented.

a Best hit from Blastx as of March 15, 2014. When the best hit was an ’unknown protein’ the best annotated hit was taken instead.

Six genes among those pinpointed by the Bingo enrichment analysis (three from the 4x > 2x group, three from the 4x < 2x group) were tested by qRT-PCR (Figure S9). Sequencing of the amplicons confirmed the identity of the genes (Table S16). The 2x–4x expression differences from the microarray experiment (Figure S10) were confirmed for all genes, and the trends were validated.

### Genome methylation

**Figure 7:**
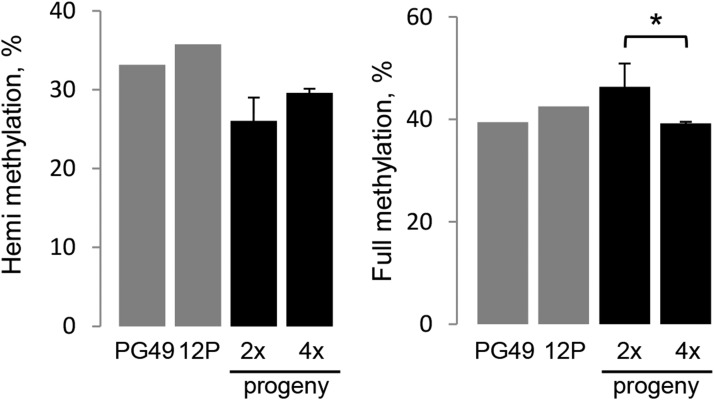
The average cytosine methylation levels in PG—F9 and 12P parents (gray bars), and their 2x and 4x hybrid hybrids (black bars). (A) Methylation changes at hemi-methylated sites. (B) methylation changes at fully methylated sites. ** Different at *P* < 0.05, Duncan’s multiple range test.

**Figure 8:**
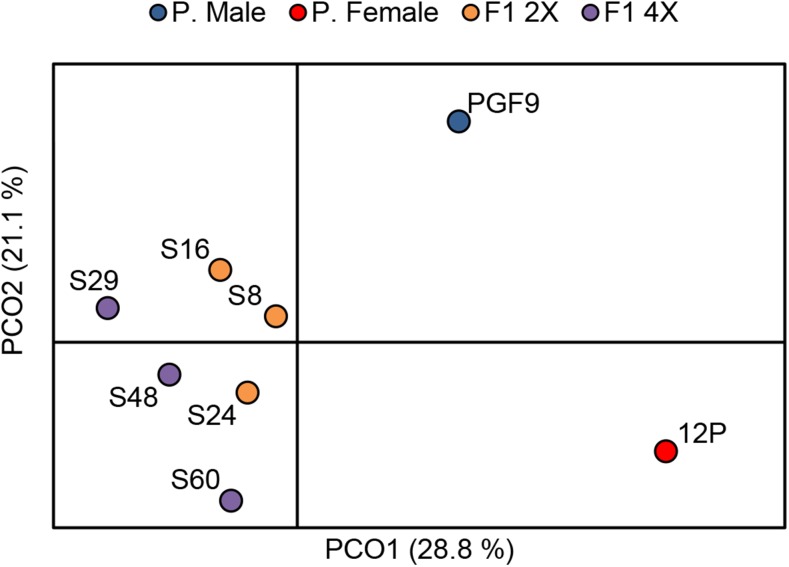
Results of principal coordinates analysis (PCA) of MSAP patterns. The first two coordinates (PCO1 and PCO2) are displayed with the indication of the percentage of variance explained in brackets. Labels indicate the centroids of each group.

When interpreting the results of MSAP analysis in our materials, it should be kept in mind that the parents are heterozygous, and segregation of parental alleles occurs both in *n* and in *2n* gametes deriving from restitutional meiosis. Therefore, the loss of a MSAP band may be a consequence not only of *de novo* cytosine methylation, but also of segregation (Aversano *et al.* 2012). On the contrary, when a band that was not present in the parents appears in a progeny plant, it can be attributed to cytosine demethylation. Based on the MSAP profiles, the number and frequency of unmethylated, hemi-methylated, and fully methylated CCGG sites were calculated for PG-F9, 12P, and their 2x and 4x hybrids (Figure 7). The 2x hybrids exhibited a higher frequency of full methylation (46.4%) than the 4x hybrids (39.2%), which did not differ from the parents. Therefore, hybridization promoted DNA full methylation at CCGG sites, and polyploidization appeared to have the opposite effect. By contrast, the differences between 2x and 4x hybrids in terms of hemi-methylation levels were not significant (Figure 7). We determined all the changes in cytosine methylation patterns between parents and each hybrid, and classified the methylation patterns into five different classes, namely: monomorphic, parental pattern inheritance, *de novo* methylation, demethylation, and unidentified/ambiguous (Table 4). In both the 2x and 4x hybrids, most cytosine methylation patterns were monomorphic (41.8% and 34.5, respectively), or were inherited from one of the parents (32.7% and 36.2%, respectively). Regarding the nonparental methylation pattern inheritances, we observed statistically significant differences (*P* < 0.05) in demethylation sites between 2x and 4x hybrids: in 4x hybrids, 15.5% of the sites lost parental methylation, *vs.* only 9.6% in diploids (Table 4). Although methylation patterns in each progeny differed among individuals, the MR values showed that *de novo* methylation was more frequent than demethylation in 2x hybrids (MR = 1.4), while the opposite was true in 4x hybrids (MR = 0.8, Table 4). Therefore, hybridization, and not polyploidization, appeared to promote DNA demethylation in our materials. Principal coordinates analysis of MSAP profiles (Figure 8) grouped the progeny plants consistently with their genetic similarity: the parents are wide apart, whereas the hybrids (which are full sibs) are closer together, without clear separation between 2x and 4x groups.

**Table 4:** Results of MSAP analysis

Genotypes	Total	Monomorphic	Parental Pattern Inheritance		Nonparental Pattern Inheritance		Ambiguous*^a^*	MR*^b^*
			PG-F9 like	12-P like	Demethylation	*De novo* Methylation		
2x progenies								
S8	221	100*^c^* (45.25*^d^*)	41 (18.55)	28 (12.67)	24 (10.86)	22 (9.95)	6 (2.71)	0.9
S16	235	101 (42.98)	54 (22.98)	21 (8.94)	26 (11.06)	27 (11.49)	6 (2.55)	1.0
S24	234	87 (37.18)	43 (18.38)	39 (16.67)	16 (6.84)	46 (19.66)	3 (1.28)	2.9
Total	690	288 (41.74)	138 (20.00)	88 (12.75)	66 (9.57) *	95 (13.77)	15 (2.17)	1.4
4x progenies								
S29	250	90 (36.00)	63 (25.20)	30 (12.00)	30 (12.00)	33 (13.20)	4 (1.60)	1.1
S48	242	79 (32.64)	50 (20.66)	42 (17.36)	42 (17.36)	28 (11.57)	1 (0.41)	0.7
S60	250	87 (34.80)	41 (16.40)	43 (17.20)	43 (17.20)	32 (12.80)	4 (1.60)	0.7
* *Total	742	256 (34.50)	154 (20.75)	115 (15.50)	115 (15.50) *	93 (12.53)	9 (1.21)	0.8

Number and percentage of methylation markers scored in each 2x or 4x hybrid in comparison with their parents. MR, methylation ratio.

a Methylation changes difficult to interpret according to Fulneček and Kovařík (2014).

b MR: methylation ratio is the percent ratio of the number of markers revealing *de novo* methylation over the number of markers revealing demethylation.

c The number of corresponding sites.

d The frequency of the corresponding sites.

* These two figures differ significantly at *P* ≤ 0.05.

## Discussion

The polyploidization system used in this study exploits spontaneous mutations of gametogenesis leading to 2*n* gamete formation, and, as such, simulates natural polyploidization events. Since the natural distribution range of subsp. *coerulea* and *falcata* overlap, and hybrids occur in nature at both 2x and 4x levels (Small 2011), a cross similar to PG-F9 × 12P could have occurred spontaneously. From this point of view, our experimental materials differ from those used in previous studies on plant neopolyploids, obtained by artificial chromosome doubling involving tissue culture or spindle-inhibiting substances. Therefore, this work offers an original perspective with which to investigate the adaptive advantages of polyploidy (Hilu 1993, Mayrose *et al.* 2011; Madlung and Wendel 2013).

### Neopolyploids show tetrasomic inheritance for most chromosomes

In alfalfa, quadrivalent pairing is infrequent (Stanford *et al.* 1972), so cytogenetic investigations are not useful to demonstrate tetrasomic inheritance; molecular marker segregation, on the contrary, can give clear evidence of pairing behavior. Cytogenetic studies have shown differences in C-banding patterns between *M. sativa* subsp. *falcata* and *M. sativa* subsp. *coerulea* (Bauchan and Hossain 1997), revealing differences in constitutive heterochromatin content of chromosomes. Therefore, preferential pairing may be expected in the BSP plants because they have two *falcata* chromosome sets from PG-F9, and two mixed *falcata* x *sativa* chromosome sets from 12P, deriving from two meiotic recombination rounds after the *falcata* x *sativa* cross. Preferential pairing implies disomic inheritance and allopolyploidy, whereas random pairing leads to tetrasomic inheritance, which is characteristic of autotetraploids (reviewed by Parisod *et al.* 2010). Therefore, the first question is whether or not our BSP plants are true autopolyploids. SSR marker segregation allowed us to answer this question: tetrasomic inheritance was the rule, with only a few exceptions, indicating that chromosome homology between *falcata* and *sativa* is high, in spite of the large morphological difference between the subspecies. Chromosome V was the only one showing a consistent tendency toward disomic segregation. Preferential pairing of this chromosome could depend on the amount and pattern of recombination between *sativa* and *falcata* chromosomes occurred in 12P (Figure S11).

### Polyploid alfalfa hybrids show phenotypic superiority and novel variation for adaptive traits

For several traits our autopolyploids performed better than both their parents and 2x hybrids, suggesting that sexual polyploidization between heterozygous, diverse genotypes resulted in increased heterozygosity of 4x BSP plants, a possible cause of “polyploid heterosis”. By contrast, in maize, tobacco, and potato autopolyploids obtained through somatic doubling, 4x plants did not display a polyploid superiority (Riddle *et al.* 2010; Stupar *et al.* 2007; Anssour *et al.* 2009; Aversano *et al.* 2015). This probably reflects the fact that somatic polyploids have the same alleles as the diploid parents, and thus do not have increased allele interactions that can enhance heterosis. Higher seed set in crosses was the most striking effect of polyploidization. It is likely that sterility factors present in parents were partly offset by complementation in the 2x hybrids, resulting in heterosis for seed set, and this complementation was much higher at the 4x level. Masking of unfavorable parental alleles as a consequence of sexual polyploidization form the basis of the high fitness of first-generation polyploids, and contribute to reproductive success. Such advantage of neopolyploids may not be uncommon (Gross and Schiestl 2015). A positive effect of chromosome doubling *per se* on fertility in alfalfa was demonstrated by Obajimi and Bingham (1973). Higher seed set would allow more abundant seed production before summer drought in the southern part of the species distribution area in which cultivation of 4x alfalfa was established. Higher seed weight has been associated with adaptation advantages of polyploids (Hahn *et al.* 2013). Such a combination of traits might also form the basis of the adoption of tetraploid *M. sativa* in agriculture. In this view, sexual polyploidization can be regarded as a key factor underpinning the agricultural adoption of alfalfa, as was proposed for wheat (Dubcovsky and Dvorak 2007). Flowering time is an important adaptive trait, and has been shown to be affected by chromosome doubling in *Arabidopsis thaliana*, with autotetraploids flowering later than diploids (Chen 2010). In our study, 4x hybrids tended to flower earlier than 2x hybrids, indicating that polyploidization may result in a shorter growth cycle. These different responses to autopolyploidization are reminiscent of the responses to hybridization: early flowering is heterotic in maize, whereas late flowering is heterotic in *Brassica napus* (Chen 2010). It can be hypothesized that alfalfa 4x hybrids experience adaptive advantages in the wild. Indeed, faster development leading to earlier flowering can be an advantage in the wild to outcompete other species. In cultivated fields, flowering time directly reflects biomass production—the most important agronomic trait of forage crops. In alfalfa, fast development is desirable to gain more harvests per year and to better compete against weeds.

### Polyploidization increases nonadditive gene transcription

In this work, gene transcription levels were compared using equal amounts of total RNA per sample. We assumed that the total amount of RNA per genome was constant or, in other words, that the total RNA per cell (transcriptome) doubled with chromosome number. Therefore, relative transcription levels per genome were compared in this work (Guo *et al.* 1996; Stupar *et al.* 2007). Since cell size increased with ploidy (Beaulieu *et al.* 2008; documented here for leaf epidermal and stomata cells), when a gene showed equal transcription levels in the 2x and 4x plants, it could be assumed that there was an increase of transcription level per cell proportional to the increase in cell size (Guo *et al.* 1996; Riddle *et al.* 2010). On the contrary, different expression between ploidy levels implies that the change in transcription was not proportional to the change in cell size.

When two individuals are crossed, some genes can show nonadditive gene expression in the hybrids, that is, the transcription level of some genes can differ significantly from the parental mean. Such nonadditivity may be at the base of heterosis (reviewed in Chen 2013). This was tested in alfalfa, by comparing the numbers of nonadditively expressed genes in heterotic *vs.* nonheterotic population hybrids (Li *et al.* 2009). The heterotic hybrids showed substantially more (4.4–7.7%) nonadditively expressed genes than the nonheterotic hybrid (0.5%). Polyploidization can affect gene expression in hybrids: in fact, nonadditivity of parental gene expression is a common feature of allopolyploids (reviewed in Jackson and Chen 2010; Chen 2013). In neo-autotetraploids, subtle gene expression changes have been found. For example, in maize, about 6% of the genes were transcriptionally affected due to transition from the 2x to the 4x state (Riddle *et al.* 2010). In potato, about 12% of 9029 genes were differentially expressed in leaves between isogenic 2x and 4x potato plants (Stupar *et al.* 2007). Here, nonadditivity was observed for a small number of genes in 2x hybrids (1.38%, in the range observed by Li *et al.* 2009), but polyploidization determined a five-fold increase of nonadditively expressed genes in 4x hybrids. Cheng *et al.* (2015) found a similar response. The fact that, in the comparison of pooled 4x hybrids with parents, underexpressed genes prevailed, whereas in comparisons between single 4x hybrids with parents, overexpressed genes prevailed, suggests that polyploidization exerted a stochastic effects on gene transcription. A few hundred differentially expressed genes were evidenced in the comparison between 2x and 4x hybrids. Since the 2x and 4x plants are full sibs, we concentrated our attention on those genes that we consider to be “polyploidization-sensitive”. Polyploidization preferentially affected the expression of genes related to energy metabolism, response to stress, and the plastid compartment. GO terms significantly enriched in the PS group of genes derived from only 25 differentially expressed genes, 15 of them upregulated, and 10 downregulated in 4x *vs.* 2x plants.

Eight photosynthesis-related genes were upregulated, six of them encode chlorophyll binding proteins. These proteins bind to chlorophyll *a* and *b* to form the light-harvesting complexes (LHC) I and II, that capture and deliver light excitation energy to photosystems I and II. The photosystem I subunit *Psa*D, and the Rubisco small subunit, were also overexpressed as a consequence of ploidy change. It can be speculated that the observed per genome increases in the expression of photosynthetic genes contributed to the increased biomass of tetraploids.

Four overexpressed lipoxygenase genes were enriched in the PS group. One of the main pathways of lipid alteration is the formation of polyunsaturated fatty acids (PUFAs) (Feussner and Wasternack 2002). Their oxidation to hydroperoxides can be catalyzed by lipoxygenases (LOX). Among plastid LOX, 13-LOX is the first enzyme of the jasmonate pathway. Jasmonates are ubiquitous lipid-derived signaling compounds active in plant development, and in plant responses to biotic and abiotic stresses (Wasternack *et al.* 2013). Increased expression of LOX in 4x plants may influence many cellular processes related to biotic and abiotic stress responses. Two heat shock protein (HSP) genes, homologous to Hsp70 (the gene with the highest fold change) and to Hsp90, respectively, were nonadditively overexpressed in 4x hybrids. These chaperone proteins are involved in the response to heat, and to many other stress factors, and their increased expression may provide advantages to the 4x condition. In *A. thaliana* allopolyploids, of 33 HSP genes, 31 were underexpressed, and three overexpressed, compared with average expression in tetraploid parents (Wang *et al.* 2006), but the comparison with diploid parents was not made.

Only 10 PS, downregulated genes contributed to the GO terms enrichment, with no obvious link between them. Among them, a mini-chromosome maintenance (MCM) protein gene got our attention. The eukaryotic replicative helicase is composed of six distinct, but related, subunits MCM(2–7). The MCM complex binds to chromatin at the site of replication initiation, and is required for chromosome replication in eukaryotes (Bell and Botchan 2013). Its release from the replication initiation site and degradation prevents reinitiation of replication in the same cell cycle. In humans, high expression of MCM protein is observed in some cancerous cells (see, for example, Ishimi *et al.* 2003). Given that, it can be speculated that reduced expression of a MCM subunit in polyploid alfalfa on a per genome basis might correlate with a decrease in the number of cell divisions in leaf tissue, contributing to the observed lower cell number, and larger cell size. Further studies are needed to test this hypothesis.

### Polyploidization does not induce DNA methylation

On average, hybridization promoted cytosine methylation, but not when combined with polyploidization. Indeed, less fully methylated sites were found in 4x than in the 2x hybrids. It is possible that, when the balanced parental genomes merge in the 4x hybrids, the resulting 4x genome is more balanced than that of the 2x hybrids, in which *de novo* methylation might be necessary to balance gene expression. Whether cytosine methylation variation is induced by hybridization or genome duplication in polyploid plants is still an open question. Madlung *et al.* (2002) and Salmon *et al.* (2005) argued that changes in cytosine methylation arise most likely as an effect of hybridization rather than genome doubling *per se*. However, Hegarty *et al.* (2011) investigated the nonadditive changes of cytosine methylation in allopolyploid *Senecio* and showed that, despite the significant effect of interspecific hybridization, polyploidization led to a secondary effect on methylation, with reversion to additivity at some loci, and novel methylation at others. Changes in cytosine methylation levels and patterns during the process of hybridization and allopolyploidization have been well documented, and our data are consistent with the findings in polyploids of *Cucumis* (Chen and Chen 2008), watermelon (Wang *et al.* 2009), sage (Li *et al.* 2012), and poplar (Suo *et al.* 2015). We found that methylation patterns differed among genotypes within 2x and 4x hybrids. Similar differences in methylation levels among individuals were reported in polyploids of *Taraxacum officinale* (Verhoeven *et al.* 2010), *Solanum* spp. (Aversano *et al.* 2013) and *Populus* (Suo *et al.* 2015). Due to instability of cytosine methylation at different genomic loci (Zhao *et al.* 2007), it may be speculated that a number of metastable loci show random alterations as a consequence of hybridization, resulting in individual differences.

### Conclusion

In a recent interesting review, Parisod *et al.* (2010), stated that: “the successful range expansion and radiation demonstrated in various natural autopolyploids suggest that genome multiplication *per se* may represent an evolutionary advantage”. In this work, we showed that sexual polyploidization conferred on alfalfa traits that can be advantageous both in the wild, and in cultivation. Several hundred genes, related to diverse metabolic functions, changed their expression level as a consequence of polyploidization. The meaning of these transcriptional changes will be investigated in further research. In addition, we found that DNA cytosine methylation was affected by both hybridization and polyploidization, suggesting that it acts as a regulatory mechanism in both events. Together, our results show that sexual polyploidization can induce phenotypic, transcriptional and DNA methylation novelties, and can form the basis of the improved adaptation and reproductive success of tetraploid *M. sativa* with respect to its diploid progenitor. Such polyploidy-induced changes may have promoted the adoption of tetraploid alfalfa in agriculture.

## 

## Supplementary Material

Supplemental Material
