# Supplementary material for: Sexual Polyploidization in Medicago sativa L.: Impact on the Phenotype, Gene Transcription, and Genome Methylation
Source: G3 (Bethesda). 2016 Feb 5;6(4):925–38. doi: 10.1534/g3.115.026021 (PMC4825662; doi:10.1534/g3.115.026021)
Supplement: Supplemental Material [file supp_6_4_925__index.html]

Sexual Polyploidization in Medicago sativa L.: Impact on the Phenotype, Gene Transcription, and Genome Methylation — Supplemental Material 

# Sexual Polyploidization in *Medicago sativa* L.: Impact on the Phenotype, Gene Transcription, and Genome Methylation

## Supplemental Material for Rosellini *et al.*, 2016

**Files in this Data Supplement:**

- Figure S1 - Scheme of the transmission of 2x parent-specific alleles by 4x plants deriving from bilateral sexual polyploidization (4x-BSP). The scheme is simplified by disregarding the 12P parent alleles. (.pdf, 102 KB)
- Figure S2 - Distribution of Blast results for the 341 Ploidy-Sensitive genes. (.pdf, 54 KB)
- Figure S3 - Species distribution of the Blast hits of Ploidy-sensitive genes. (.pdf, 92 KB)
- Table S1 - Primers for the amplification of the selected, informative SSR markers. (.pdf, 13 KB)
- Table S9 - Dry biomass yield of 2x and 4x hybrids and their parents. (.pdf, 18 KB)
- Table S10 - Dry biomass percentage of 2x and 4x hybrids and their parents. (.pdf, 18 KB)
- Table S11 - Flowering time. (.pdf, 18 KB)
- Table S12 - Fertility traits. (.pdf, 20 KB)
- Table S13 - Number of differentially expressed genes in single-genotype comparisons. (.pdf, 14 KB)
- Table S14 - GO terms significantly enriched in the PS genes with respect to the genes present in the microarray, according to BinGO analysis. (.pdf, 32 KB)
- Table S15 - Primers used for qRT-PCR analysis of six random transcripts. (.pdf, 83 KB)
- Figure S4 - Distribution of GO terms for the Biological function vocabulary among the 341 Ploidy-sensitive genes. (.pdf, 197 KB)
- Table S16 - Pairwise alignments between amplicon sequences of the *M. sativa* genes tested by qRT-PCR analysis and the *M. truncatula* TCs used for microarray and primer design (.pdf, 106 KB)
- Table S2 - Allelic configurations of SSR markers that were tested and selected (shaded) for the analysis of chromosome pairing behavior. (.pdf, 31 KB)
- Table S3 - X2 analysis of segregation mode of the BSP plant S29-4x. (.pdf, 20 KB)
- Table S4 - X2 analysis of segregation mode of the BSP plant S48-4x. (.pdf, 22 KB)
- Table S5 - X2 analysis of segregation mode of the BSP plant S60-4x. (.pdf, 21 KB)
- Table S6 - Number of alleles in parents and 4x BSP hybrids. (.pdf, 13 KB)
- Table S7 - Leaf morphology traits of 2x and 4x hybrids and their parents. (.pdf, 21 KB)
- Table S8 - Fresh biomass yield of 2x and 4x hybrids and their parents. (.pdf, 19 KB)
- Figure S5 - Distribution of GO terms for the Cellular component vocabulary among the 341 Ploidy-sensitive genes. (.pdf, 58 KB)
- Figure S6 - Distribution of GO terms for the Molecular function vocabulary among the 341 Ploidy-sensitive genes. (.pdf, 117 KB)
- Figure S7 - Distribution of GO terms for the Biological process vocabulary among the 341 Ploidy-sensitive genes, adopting the Goslim option for plants. (.pdf, 112 KB)
- Figure S8 - Distribution of GO terms for the Cellular component vocabulary among the 341 Ploidy-sensitive genes, adopting the Goslim option for plants. (.pdf, 64 KB)
- Figure S9 - Distribution of GO terms for the Molecular function vocabulary among the 341 Ploidy-sensitive genes, adopting the Goslim option for plants. (.pdf, 85 KB)
- Figure S10 - Comparison of transcriptional levels of six genes among those evidenced by Bingo analysis, as measured by qRT-PCR (A, B) and by microarrays (C, D). (.pdf, 131 KB)
- Figure S11 - Model of possible structures of a the four homologous chromosomes in two 4x BSP plants from the cross PG-F9 x 12P. (.pdf, 28 KB)
- Table S17 - Normalized expression and standard deviation for probe sets that are differentially expressed between parental and progeny means, and fold change and significance (false discovery rate) of each paired comparison.(.xls, 716 KB)
