# Supplementary material for: Sexual Polyploidization in Medicago sativa L.: Impact on the Phenotype, Gene Transcription, and Genome Methylation
Source: G3 (Bethesda). 2016 Feb 5;6(4):925–38. doi: 10.1534/g3.115.026021 (PMC4825662; doi:10.1534/g3.115.026021)
Supplement: Supplemental Material [file supp_g3.115.026021_FigureS1.pdf]

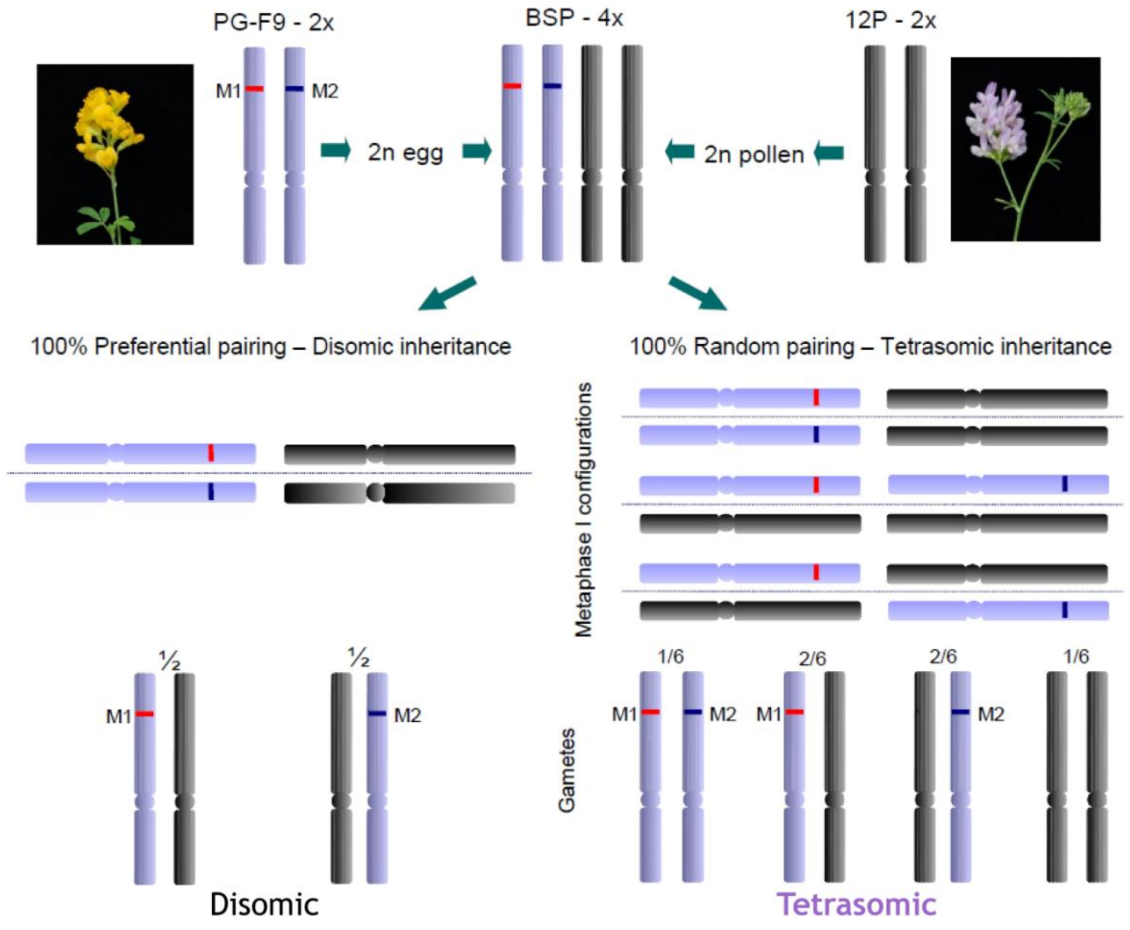

Figure S1. Scheme of the transmission of 2x parent-specific alleles by 4x plants deriving from bilateral sexual polyploidization (4x-BSP). The scheme is simplified by disregarding the 12P parent alleles. One or both alleles from either 2x parent can be used for the segregation analysis, if absent in the 4x partner of the test cross 4x-BSP x 4x tester (not represented).
