# Supplementary figures and images for: Sexual Polyploidization in Medicago sativa L.: Impact on the Phenotype, Gene Transcription, and Genome Methylation
Source: G3 (Bethesda). 2016 Feb 5;6(4):925–38. doi: 10.1534/g3.115.026021 (PMC4825662; doi:10.1534/g3.115.026021)

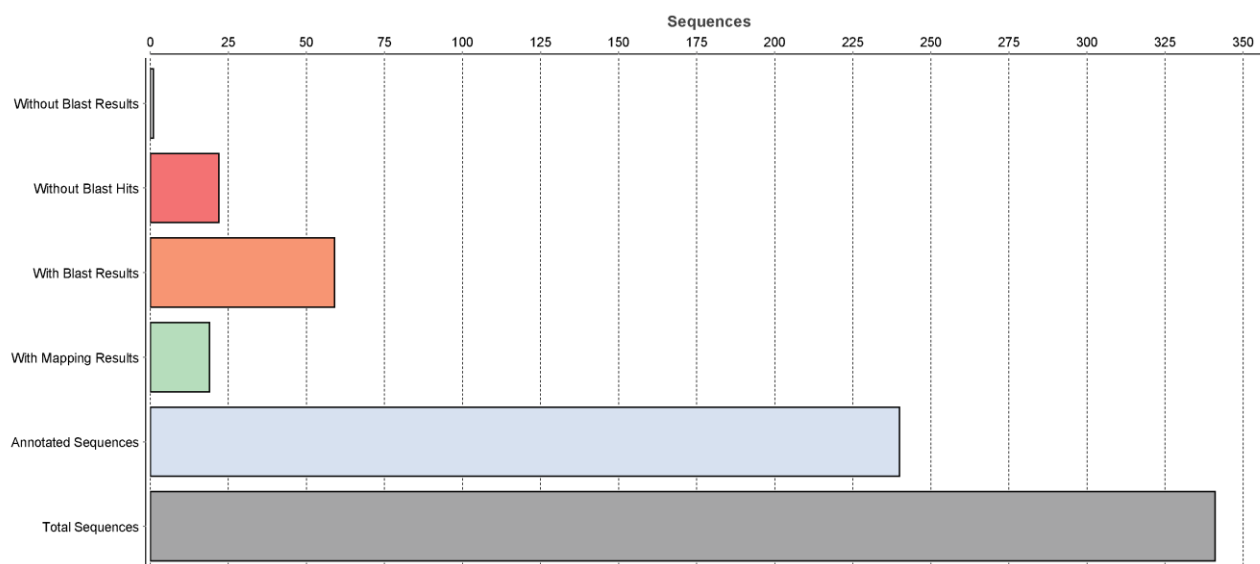

**Figure S2. Distribution of Blast results for the 341 Ploidy-Sensitive genes**

Supplement: Supplemental Material [file supp_g3.115.026021_FigureS2.pdf]

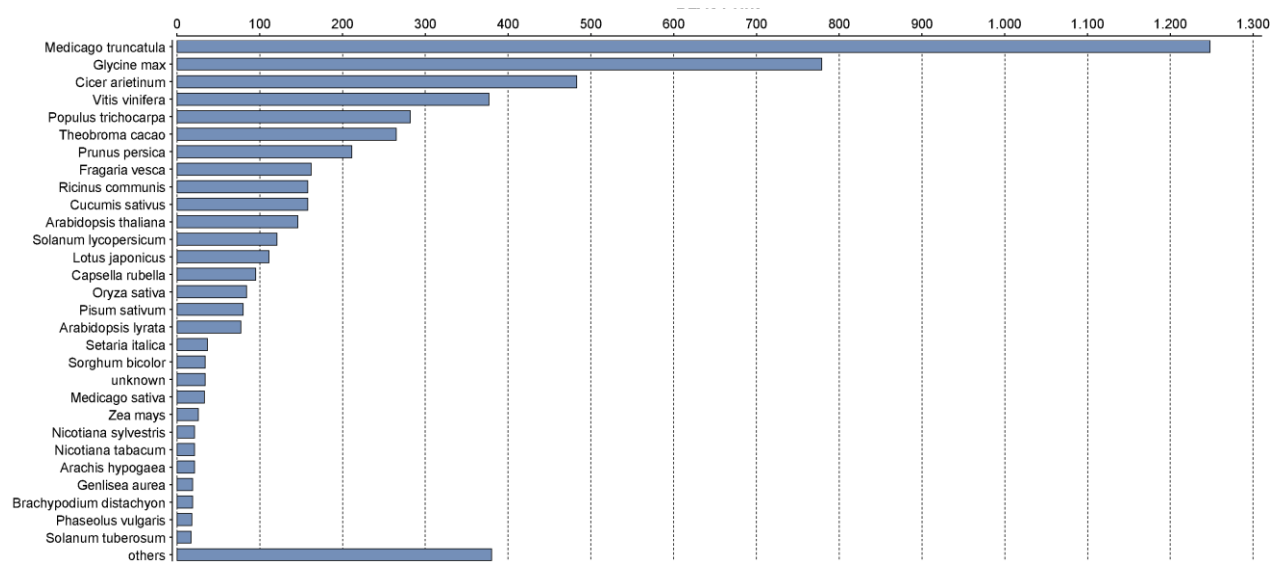

**Figure S3. Species distribution of the Blast hits of Ploidy-sensitive genes.**

Supplement: Supplemental Material [file supp_g3.115.026021_FigureS3.pdf]
