# Supplementary material for: Sexual Polyploidization in Medicago sativa L.: Impact on the Phenotype, Gene Transcription, and Genome Methylation
Source: G3 (Bethesda). 2016 Feb 5;6(4):925–38. doi: 10.1534/g3.115.026021 (PMC4825662; doi:10.1534/g3.115.026021)
Supplement: Supplemental Material [file supp_g3.115.026021_TableS1.pdf]

Table S1. Primers for the amplification of the selected, informative SSR markers (from Diwan *et al*. 2000, Julier *et al*. 2003; Sledge *et al.* 2005; Mun *et al*. 2006)

Marker	Chromo-some	Primer sequences		Annealing Temp.
FMT13	I	GATGAGAAAATGAAAAGAAC	CAAAAACTCACTCTAACACAC	50
MTIC451	II	GGACAAAATTGGAAGAAAAA	AATTACGTTTGTTTGGATGC	55
MTIC189	III	CAAACCCTTTTCAATTTCAACC	ATGTTGGTGGATCCTTCTGC	59
MTIC332	IV	CCCTGGGTTTTTGATCCAG	GGTCATACGAGCTCCTCCAT	60
B14B03	V	GCTTGTTCTTCTTCAAGCTC	ACCTGACTTGTGTTTTATGC	56
MTIC48	V	TTTTTGTTAGTTTGATTTTAGGTG	GCTACAAAGTCTTCTTCCACA	56
MTIC153	VI	TCACAACTATGCAACAAAAGTGG	TGGGTCGGTGAATTTTCTGT	59
MTIC273(1)	VII	TGTTAGCAACTTTGTGATGG	TCCATTACAATACCCAGAGG	54
MTIC135	VIII	GCTGACTGGACGGATCTGAG	CCAAAGCATAAGCATTCATTCA	60
