# Supplementary material for: Sexual Polyploidization in Medicago sativa L.: Impact on the Phenotype, Gene Transcription, and Genome Methylation
Source: G3 (Bethesda). 2016 Feb 5;6(4):925–38. doi: 10.1534/g3.115.026021 (PMC4825662; doi:10.1534/g3.115.026021)
Supplement: Supplemental Material [file supp_g3.115.026021_TableS9.pdf]

Table S9. **Dry biomass yield of 2x and 4x hybrids and their parents. Means followed by different letters are significantly different at *P*<0.05.**

**Plant**	**Ploidy**	**[1] Dry matter**		
**PARENTS**		**1st cut**	**2nd cut**	**3rd cut**
PG-F9	2x	15.38	11.26	8.65
12P		12.49	12.00	8.21
**Parental mean**		**13.31**	**11.78 C**	**8.34**
**HYBRIDS**				
S8	2x	11.84	14.34	7.59
S16		21.53	13.82	9.72
S24		13.02	23.16	15.38
**2x hybrids mean**		**15.46**	**17.89 B**	**11.37**
S29	4x	15.49	24.58	14.13
S48		14.70	23.05	12.42
S60		13.44	25.31	9.84
**4x hybrids mean**		**14.54**	**24.11 A**	**12.59**

[1] Average of 2-6 rooted cuttings per genotype.
