# Supplementary material for: Sexual Polyploidization in Medicago sativa L.: Impact on the Phenotype, Gene Transcription, and Genome Methylation
Source: G3 (Bethesda). 2016 Feb 5;6(4):925–38. doi: 10.1534/g3.115.026021 (PMC4825662; doi:10.1534/g3.115.026021)
Supplement: Supplemental Material [file supp_g3.115.026021_TableS10.pdf]

Table S10. **Dry biomass percentage of 2x and 4x hybrids and their parents. Means followed by different letters are significantly different at *P*<0.05.**

**Plant**	**Ploidy**	**(1) Dry mass %**			
**PARENTS**		**1st cut**	**2nd cut**	**3rd cut**	
PGF9	2x	24.00	26.00	28.67	
12P		24.20	25.29	24.43	
**Parental mean**		**24.14 A**	**25.50 B**	**25.70**	
**HYBRIDS**					
S8	2x	22.60	29.25	29.00	
S16		22.20	28.67	28.00	
S24		20.60	31.60	30.60	
**2x hybrids mean**		**21.80 B**	**30.08 A**	**29.42**	
S29	4x	22.20	31.50	28.25	
S48		20.20	30.75	28.25	
S60		21.20	33.50	30.00	
**4x hybrids mean**		**21.20 B**	**31.60 A**	**28.60**	

(1) Average of 2-6 rooted cuttings per genotype. Means in a column followed by different letters differ significantly according to ANOVA and LSMEANS (capital letters) or by LSMEANS only (small case letters)
