# Supplementary material for: Sexual Polyploidization in Medicago sativa L.: Impact on the Phenotype, Gene Transcription, and Genome Methylation
Source: G3 (Bethesda). 2016 Feb 5;6(4):925–38. doi: 10.1534/g3.115.026021 (PMC4825662; doi:10.1534/g3.115.026021)
Supplement: Supplemental Material [file supp_g3.115.026021_TableS11.pdf]

Table S11. **Flowering time**

Plant	Ploidy	Flowering time [3]	
**PARENTS**		**First regrowth [1]**	**Second regrowth [2]**
PG-F9	2x	35.33	50.33
12P		36.71	29.29
**Parental mean**		**36.30 B**	**35.60 B**
**HYBRIDS**			
S8	2x	40.75	46.00
S16		42.00	49.00
S24		40.60	37.40
**2x hybrids mean**		**41.00 A**	**43.17 A**
S29	4x	40.00	39.75
S48		37.50	35.25
S60		41.50	40.00
**4x hybrids mean**		**39.30 AB**	**38.00 AB**

(1) Days from June 1st

(2) Days from previous cut.

(3) Within a column, Means followed by different letters are significantly different at $P<0.05$
