# Supplementary material for: Sexual Polyploidization in Medicago sativa L.: Impact on the Phenotype, Gene Transcription, and Genome Methylation
Source: G3 (Bethesda). 2016 Feb 5;6(4):925–38. doi: 10.1534/g3.115.026021 (PMC4825662; doi:10.1534/g3.115.026021)
Supplement: Supplemental Material [file supp_g3.115.026021_TableS12.pdf]

Table S12. **Fertility traits. Seed set is from hand-crossing without emasculation. Means followed by different letters are significantly different at *P*≤0.05, according to the LSD test.**

Plant	Ploidy	Ovule number(1)	Ovule sterility (2)	Pollen diameter (3)	Seed weight(4)	Seed set in hand crosses (5)		
						Unrelated 2x Pollen parent (6)	Unrelated 4x Pollen parent (6)	Between full sibs
**PARENTS**								
PG-F9	2x	9.50	21.0	34.0	1.67	0.61	0.45	-
12-P		12.50	45.3	32.0	1.43	0.24	0.00	-
**Parental mean**		**11.07 A**	**33.7 A**	**32.7 B**	**1.55 B**	**0.43 B**	**0.23**	**-**
**HYBRIDS**								
S8		8.40	30.3	32.0	1.67	0.43	0.26	0.85
S16	2x	10.32	47.6	32.1	1.20	0.62	0.16	0.29
S24		9.05	32.2	32.7	1.32	1.17	0.15	0.86
**2x Hybrids mean**		**9.28 B**	**36.9 A**	**32.2 B**	**1.40 B**	**0.74 B**	**0.19**	**0.67 A**
S29		10.68	19.8	38.6	2.17	0.00	0.99	0.15
S48	4x	8.67	9.4	38.6	2.16	0.02	2.17	0.26
S60		9.40	24.1	40.9	2.05	0.00	2.20	0.06
**4x Hybrids mean**		**9.60 B**	**17.7 B**	**39.4 A**	**2.13 A**	**0.01**	**1.79 A**	**0.16 B**

(1) Number of ovules per ovary, based on 20-22 random florets per plant.
(2) Percentage of ovules showing massive callose deposition per pistil, based on twenty random pistils per plant.
(3) µ, based on 30-55 pollen grains per plant.
(4) Based on 23 to 291 seeds per plant, obtained from crosses with unrelated pollen parents of the same ploidy
(5) Means of four racemes (replications) in each of two environments, each with 8-30 florets cross-pollinated by hand without emasculation.
(6) Statistical comparison was made excluding interploidy crosses. The 2x hybrids appear to have inherited the 2n egg trait from PG-F9, because they produced seed in 2x-4x crosses, with relatively high expression in S8.
