# Supplementary material for: Sexual Polyploidization in Medicago sativa L.: Impact on the Phenotype, Gene Transcription, and Genome Methylation
Source: G3 (Bethesda). 2016 Feb 5;6(4):925–38. doi: 10.1534/g3.115.026021 (PMC4825662; doi:10.1534/g3.115.026021)
Supplement: Supplemental Material [file supp_g3.115.026021_TableS13.pdf]

Table S13. **Number of differentially expressed genes in single-genotype comparisons. For each comparison, the three numbers correspond, respectively, to the genes with higher expression in the genotypes reported in the line headings, to the genes with higher expression in the genotypes reported in the column headings, and the total number (and percentages) of differentially expressed genes. Shadings designate the comparisons between parents (light blue), 2x (gray) and 4x hybrids (light yellow)**

**Genotypes**	**12P**	**S8 2x**	**S16 2x**	**S29 4x**	**S48 4x**
**PG-F9**	2561, 2278<br>4839 (11.65%)	-	-	-	-
**S16 2x**	-	803, 639<br>1442 (3.47%)	-	-	-
**S24 2x**	-	885, 829<br>1714 (413%)	907, 778<br>1685 (4.06%)	-	-
**S48 4x**	-	-	-	574, 1010<br>1584 (3.81%)	-
**S60 4x**	-	-	-	414, 357<br>771 (1.86%)	892, 480<br>1372 (3.30%)
