# Supplementary material for: Sexual Polyploidization in Medicago sativa L.: Impact on the Phenotype, Gene Transcription, and Genome Methylation
Source: G3 (Bethesda). 2016 Feb 5;6(4):925–38. doi: 10.1534/g3.115.026021 (PMC4825662; doi:10.1534/g3.115.026021)
Supplement: Supplemental Material [file supp_g3.115.026021_TableS14.pdf]

Table S14. GO terms significantly enriched in the PS genes with respect to the genes present in the microarray, according to BinGO analysis

GO-ID	corrected p-value	X[(1)]	n[(1)]	X[(1)]	N[(1)]	Description	Genes in test set
						Molecular function	
16165	0.0000	5	13	60	9590	linoleate 13S-lipoxygenase activity	TC201583|TC173777|TC185299|TC172508|TC178639
16168	0.0000	6	34	60	9590	chlorophyll binding	TC188202|TC195187|TC179482|TC183902|TC180095|TC177786
46906	0.0000	6	39	60	9590	tetrapyrrole binding	TC188202|TC195187|TC179482|TC183902|TC180095|TC177786
16701	0.0000	5	21	60	9590	oxidoreductase activity, acting on single donors with incorporation of molecular oxygen	TC201583|TC173777|TC185299|TC172508|TC178639
16702	0.0000	5	21	60	9590	oxidoreductase activity, acting on single donors with incorporation of molecular oxygen, incorporation of two atoms of oxygen	TC201583|TC173777|TC185299|TC172508|TC178639
51213	0.0011	5	62	60	9590	dioxygenase activity	TC201583|TC173777|TC185299|TC172508|TC178639
						Biological process	
9769	0.0000	6	23	61	8883	photosynthesis, light harvesting in photosystem II	TC188202|TC195187|TC179482|TC183902|TC180095|TC177786
9765	0.0001	6	37	61	8883	photosynthesis, light harvesting	TC188202|TC195187|TC179482|TC183902|TC180095|TC177786
30397	0.0001	4	11	61	8883	membrane disassembly	TC201583|TC173777|TC172508|TC178639
9816	0.0007	4	17	61	8883	defense response to bacterium, incompatible interaction	TC201583|TC173777|TC172508|TC178639
9694	0.0017	4	23	61	8883	jasmonic acid metabolic process	TC201583|TC173777|TC172508|TC178639
9695	0.0017	4	23	61	8883	jasmonic acid biosynthetic process	TC201583|TC173777|TC172508|TC178639
42742	0.0022	7	129	61	8883	defense response to bacterium	TC201583|TC179158|TC173777|TC182218|TC176166|TC172508|TC178639
51707	0.0022	12	429	61	8883	response to other organism	TC201583|TC179350|TC172620|TC179158|TC173777|TC175139|TC185299|TC182218|TC176166|TC172508|TC178665|TC178639
9607	0.0027	12	442	61	8883	response to biotic stimulus	TC201583|TC179350|TC172620|TC179158|TC173777|TC175139|TC185299|TC182218|TC176166|TC172508|TC178665|TC178639
19684	0.0038	6	100	61	8883	photosynthesis, light reaction	TC188202|TC195187|TC179482|TC183902|TC180095|TC177786
9617	0.0065	8	218	61	8883	response to bacterium	TC201583|TC172620|TC179158|TC173777|TC182218|TC176166|TC172508|TC178639
15979	0.0065	7	164	61	8883	photosynthesis	TC174046|TC188202|TC195187|TC179482|TC183902|TC180095|TC177786
45087	0.0070	5	74	61	8883	innate immune response	TC201583|TC179158|TC173777|TC172508|TC178639
22411	0.0070	4	40	61	8883	cellular component disassembly	TC201583|TC173777|TC172508|TC178639
6952	0.0080	10	370	61	8883	defense response	TC201583|TC179350|TC179158|TC173777|TC183565|TC175139|TC182218|TC176166|TC172508|TC178639
9814	0.0116	4	47	61	8883	defense response, incompatible interaction	TC201583|TC173777|TC172508|TC178639
51704	0.0208	12	588	61	8883	multi-organism process	TC201583|TC179350|TC172620|TC179158|TC173777|TC175139|TC185299|TC182218|TC176166|TC172508|TC178665|TC178639
9751	0.0470	4	70	61	8883	response to salicylic acid stimulus	TC200070|TC179158|TC185299|TC186568

Table S14, continued

GO-ID	corrected p-value	x	n	X	N	Description	Genes in test set
						Cellular component	
30076	0.0000	6	44	67	10863	light-harvesting complex	TC188202|TC195187|TC179482|TC183902|TC180095|TC177786
10287	0.0002	6	71	67	10863	plastoglobule	TC188202|TC195187|TC179482|TC183902|TC180095|TC177786
9570	0.0002	8	162	67	10863	chloroplast stroma	TC188202|TC198142|TC195187|TC179482|TC176016|TC183902|TC180095|TC177786
9532	0.0003	8	174	67	10863	plastid stroma	TC188202|TC198142|TC195187|TC179482|TC176016|TC183902|TC180095|TC177786
34357	0.0133	8	307	67	10863	photosynthetic membrane	TC174046|TC188202|TC198142|TC195187|TC179482|TC183902|TC180095|TC177786
44436	0.0154	8	335	67	10863	thylakoid part	TC174046|TC188202|TC198142|TC195187|TC179482|TC183902|TC180095|TC177786
9579	0.0154	8	345	67	10863	thylakoid	TC174046|TC188202|TC198142|TC195187|TC179482|TC183902|TC180095|TC177786
9535	0.0154	7	267	67	10863	chloroplast thylakoid membrane	TC188202|TC198142|TC195187|TC179482|TC183902|TC180095|TC177786
55035	0.0154	7	267	67	10863	plastid thylakoid membrane	TC188202|TC198142|TC195187|TC179482|TC183902|TC180095|TC177786
42651	0.0154	7	270	67	10863	thylakoid membrane	TC188202|TC198142|TC195187|TC179482|TC183902|TC180095|TC177786
9534	0.0201	7	292	67	10863	chloroplast thylakoid	TC188202|TC198142|TC195187|TC179482|TC183902|TC180095|TC177786
31976	0.0201	7	292	67	10863	plastid thylakoid	TC188202|TC198142|TC195187|TC179482|TC183902|TC180095|TC177786
31984	0.0209	7	298	67	10863	organelle subcompartment	TC188202|TC198142|TC195187|TC179482|TC183902|TC180095|TC177786
44434	0.0277	8	405	67	10863	chloroplast part	TC188202|TC198142|TC195187|TC179482|TC176016|TC183902|TC180095|TC177786
44435	0.0361	8	428	67	10863	plastid part	TC188202|TC198142|TC195187|TC179482|TC176016|TC183902|TC180095|TC177786
42555	0.0451	1	1	67	10863	MCM complex	TC173466

(1) x, occurrence of each GO term in the test set; n, occurrence of each GO term in the reference set; X, number of annotated genes in the test set (PS genes); N, number of annotated genes in the reference set
