# Supplementary material for: Sexual Polyploidization in Medicago sativa L.: Impact on the Phenotype, Gene Transcription, and Genome Methylation
Source: G3 (Bethesda). 2016 Feb 5;6(4):925–38. doi: 10.1534/g3.115.026021 (PMC4825662; doi:10.1534/g3.115.026021)
Supplement: Supplemental Material [file supp_g3.115.026021_TableS15.pdf]

Table S15. Primers used for qRT-PCR analysis of six random transcripts.

Gene	Forward primer	Reverse primer	Annealing Temp.°C
TC172620	CTTGGTGCTGCGAGATGTTA	AGGCGCAAGAGGTACTTTGA	58
TC198142	CGAGACCGAGAAAGGATTTG	AGGCAGTAGGGTAAGCAGCA	60
TC180095	AGATGGGCTATGTTGGGTGC	GCATGGACTAGGCTTGGGTT	60
TC176166	GTTGCACTTTCAGGTGCTCA	AGGTCCACCATTGGGACATA	58
TC200070	GAGAAAGAGCGCGAGAAGAA	TGCAGCTCGGTTATGTCTTG	60
TC173466	ATCTCTTCAGGCACGCTGTT	CAAACTTCGCAAGCATTTCA	62
β-actin	ATGTATGTGGCCATCCAGGCTG	TCCAAACGAAGGATGGCATGTG	62
