# Supplementary material for: Sexual Polyploidization in Medicago sativa L.: Impact on the Phenotype, Gene Transcription, and Genome Methylation
Source: G3 (Bethesda). 2016 Feb 5;6(4):925–38. doi: 10.1534/g3.115.026021 (PMC4825662; doi:10.1534/g3.115.026021)
Supplement: Supplemental Material [file supp_g3.115.026021_TableS16.pdf]

Table S16. Pairwise alignments between amplicon sequences of the *M. sativa* genes tested by qRT-PCR analysis and the *M. truncatula* TCs used for microarray and primer design

---

TC172620
XP_003622001.1 - Heat shock protein Hsp70, *Medicago truncatula*

TACCTTTAGTCCCAATTAAGGTTTATGAAGGTGAGAGAATTATTGCGAGTGAGAACAACTTA
CTTGGTTTGTTTAAGGTCAAAGTACCTCTTGCGCCTA

Pairwise alignment

```
>TC172620 Heat shock protein Hsp70

Score       Expect  Identities  Gaps      Strand
91.5
bits(100)   5e-23   74/90(82%)  0/90(0%)  Plus/Plus

Query  9     GTCCCAATTAAGGTTTATGAAGGTGAGAGAATTATTGCGAGTGAGAACAACTTACTTGGT  68
             ||| || || | ||||| || |||||||||  | |||| ||||||||||| || ||||||
Sbjct  1333  GTCTCAGTTGATGTTTACGAGGGTGAGAGATCTGTTGCTAGTGAGAACAATTTGCTTGGT  1392

Query  69    TTGTTTAAGGTCAAAGTACCTCTTGCGCCT  98
             || ||| |  ||||||||||||||||||||
Sbjct  1393  TTATTTGAACTCAAAGTACCTCTTGCGCCT  1422
```

TC198142
XP_003636970.1 - Ribulose bisphosphate carboxylase small chain 3A, *M. truncatula*

TCGAGACCGAGAAAGGATTTGTGTACCGTGAGAACCACAAGTCACCAGGATACTATGACGGA
CGTTACTGGACAATGTGGAAGTTGCCTTTGTTTGGAGCAACCGATTCTTCTCAAGTGTTGAAG
GAGCTTGATGAAGCTATTGCTGCTTACCCTACTG

Pairwise alignment
TC198142 similar to UniRef100_Q9ZP07 Cluster: Ribulose 1,5-bisphosphate carboxylase small subunit precursor; n=1; Cicer arietinum|Rep: Ribulose 1,5-bisphosphate carboxylase small subunit precursor - Cicer arietinum (Chickpea) (Garbanzo), partial (98%)

```
Score           Expect Identities      Gaps        Strand
260 bits(288)   3e-74  153/159(96%)    0/159(0%)   Plus/Plus

Query  1    TCGAGACCGAGAAAGGATTTGTGTACCGTGAGAACCACAAGTCACCAGGATACTATGACG  60
            |||||||||||||||||||||||||||||||||||||||  |||||||||||||||||||
Sbjct  377  TCGAGACCGAGAAAGGATTTGTGTACCGTGAGAACCACAGTTCACCAGGATACTATGACG  436

Query  61   GACGTTACTGGACAATGTGGAAGTTGCCTTTGTTTGGAGCAACCGATTCTTCTCAAGTGT  120
            ||||||||||||||||||||||||||||||||||||||||||| ||| ||||||||||||
Sbjct  437  GACGTTACTGGACAATGTGGAAGTTGCCTTTGTTTGGAGCAACTGATGCTTCTCAAGTGT  496

Query  121  TGAAGGAGCTTGATGAAGCTATTGCTGCTTACCCTACTG  159
            |||||||||||||||||| | ||||||||||||||||||
Sbjct  497  TGAAGGAGCTTGATGAAGTTGTTGCTGCTTACCCTACTG  535
```

TC180095
XP_003618482.1 - Chlorophyll a/b binding protein, *M. truncatula*

TCTTATCTCGCATGGTGTCAAATTCGGCGAGGCTGTATGGTTCAAGGCAGGATCCCAAATTT
TTAGTGAGGGTGGGCTTGACTACTTGGGTAACCCAAGCCTAGTCCATGCA

Pairwise alignment

TC180095 homologue to UniRef100_Q43437 Cluster: Photosystem II type I chlorophyll a/b-binding protein precursor; n=1; Glycine max|Rep: Photosystem II type I chlorophyll a/b-binding protein precursor - Glycine max (Soybean), partial (77%)

```
Score            Expect Identities      Gaps        Strand
159 bits(176)  5e-44  104/113(92%)    1/113(0%)   Plus/Plus

Query  1    TCTTATCTCGCA-TGGTGTCAAATTCGGCGAGGCTGTATGGTTCAAGGCAGGATCCCAAA  59
            |||| ||||||| |||||| ||||||||||||||||| ||||||||||||||||| ||||
Sbjct  407  TCTTGTCTCGCAATGGTGTGAAATTCGGCGAGGCTGTGTGGTTCAAGGCAGGATCTCAAA  466

Query  60   TTTTTAGTGAGGGTGGGCTTGACTACTTGGGTAACCCAAGCCTAGTCCATGCA  112
            | ||||||||||| || |||||||||||||||||||||||||||||||| |||
Sbjct  467  TCTTTAGTGAGGGAGGACTTGACTACTTGGGTAACCCAAGCCTAGTCCACGCA  519
```

TC176166
XP_003594497.1 - Peroxidase, *M. truncatula*

TCTTCGTTGATCGATTATACAATTTCAGCAATACTGGAAATCCCGATCCAACTCTCAACACA
ACCTACTTACAAACATTGAGAACAATATGTCCCAATGGTG

Pairwise alignment
TC176166 homologue to UniRef100_Q40366 Cluster: Peroxidase precursor; n=1; Medicago sativa|Rep: Peroxidase precursor - Medicago sativa (Alfalfa), complete

```
Score            Expect Identities      Gaps        Strand
179 bits(198)  9e-50  101/102(99%)    0/102(0%)   Plus/Plus

Query  1    TCTTCGTTGATCGATTATACAATTTCAGCAATACTGGAAATCCCGATCCAACTCTCAACA  60
            ||||||||||||||||||||||||||||||||||||||||||||||||||||||||||||
Sbjct  651  TCTTCGTTGATCGATTATACAATTTCAGCAATACTGGAAATCCCGATCCAACTCTCAACA  710

Query  61   CAACCTACTTACAAACATTGAGAACAATATGTCCCAATGGTG  102
            ||||||||||||||||||||||||| ||||||||||||||||
Sbjct  711  CAACCTACTTACAAACATTGAGAACTATATGTCCCAATGGTG  752
```

TC200070
XP_003623202.1 - Glutathione S-transferase GST 8, *M. truncatula*

GCTCTTCCTTTCTTGAGAATGAGCTGAAGGACAAGTACTTTGGCGGAGAAGAGTTTAACTTT
GTCGATATTGCGGCTGTTTTCGTCGCATTTTGGGTCCCTCTAGTTCAAGACAAACCGAGCTGC
AA

Pairwise alignment
TC200070 similar to UniRef100_Q9FQF0 Cluster: Glutathione S-transferase GST 8; n=1; Glycine max|Rep: Glutathione S-transferase GST 8 - Glycine max (Soybean), partial (98%)

```
Score            Expect Identities      Gaps        Strand
203 bits(224)  7e-57  124/129(96%)    2/129(1%)   Plus/Plus

Query  1    GCTCTTC-CTTTCTTGAGAATGAGCTGAAGGACAAGTACTTTGGCGGAGAAGAGTTTAAC  59
            ||||||| |||||||||||||||||||||||||||||||||||| |||||||||||||||
Sbjct  442  GCTCTTCACTTTCTTGAGAATGAGCTGAAGGACAAGTACTTTGGAGGAGAAGAGTTTAAC  501

Query  60   TTTGTCGATATTGCGGCTGTTTTCGTCGCATTTTGGGTCCCTCTAGTTCAAGACA-AACC  118
            |||||||||||||| ||||||||||| |||||||||||||||||||||||||||| ||||
Sbjct  502  TTTGTCGATATTGCTGCTGTTTTCGTAGCATTTTGGGTCCCTCTAGTTCAAGACATAACC  561

Query  119  GAGCTGCAA  127
            |||||||||
Sbjct  562  GAGCTGCAA  570
```

TC173466
XP_003609791.1 - DNA replication licensing factor mcm2, *M. truncatula*

CGCAGAGGATATCGAAACGAGAAATAATAGGATCTGTCAATTCAACGTTTTGAGTAAACAA
TTTTGATGAATCATATCTCCCTCCAATTGGATTGGCAGCAGCAATGACAGAACAGCGTGCCT
GAAGA

Pairwise alignment

TC173466 MCM; Nucleic acid-binding, OB-fold

```
Score           Expect Identities      Gaps         Strand
223 bits(246)   2e-62  125/126(99%)    0/126(0%)    Plus/Minus

Query  3     CAGAGGATATCGAAACGAGAAATAATAGGATCTGTCAATTCAACGTTTTGAGTAAACAAT  62
             ||||||||||||||||||||||||||||||||||||||||||||||||||||||||||||
Sbjct  2056  CAGAGGATATCGAAACGAGAAATAATAGGATCTGTCAATTCAACGTTTTGAGTAAACAAT  1997

Query  63    TTTGATGAATCATATCTCCCTCCAATTGGATTGGCAGCAGCAATGACAGAACAGCGTGCC  122
             |||||||||||||||||| |||||||||||||||||||||||||||||||||||||||||
Sbjct  1996  TTTGATGAATCATATCTTCCTCCAATTGGATTGGCAGCAGCAATGACAGAACAGCGTGCC  1937

Query  123   TGAAGA  128
             ||||||
Sbjct  1936  TGAAGA  1931
```
