# Supplementary material for: Sexual Polyploidization in Medicago sativa L.: Impact on the Phenotype, Gene Transcription, and Genome Methylation
Source: G3 (Bethesda). 2016 Feb 5;6(4):925–38. doi: 10.1534/g3.115.026021 (PMC4825662; doi:10.1534/g3.115.026021)
Supplement: Supplemental Material [file supp_g3.115.026021_TableS2.pdf]

Table S2. **Allelic configurations of SSR markers that were tested and selected (shaded) for the analysis of chromosome pairing behavior. Figures are the sizes in bp of the SSR amplicons.**

**Marker, Chromosome**	**Alleles**	**PGF9 (2x)**	**12P (2x)**	**CLASSE (4x)**	**BSP S29 (4x)**	**BSP S48 (4x)**	**BSP S60 (4x)**
FMT13, I	M1		144.33	144.71	144.46	144.59	144.59
	M2		146.93	147.05	147.05	147.05	147.05
	M3	151.75			151.74	151.86	151.74
	M4			155.86			
	M5			165.59			
MTIC451, II	M1	124.02				124.16	124.09
	M2		130.05	130.03	130.05		130.02
	M3			134.05			
	M4	136.14			136.04	135.93	
	M5		137.99	137.99	138.09	138.09	138
MTIC189, III	M1		112.72	112.76	112.85	112.8	112.69
	M2	118.69			118.73		118.65
	M3			124.8			
	M4	131.01				131.1	131.04
	M5		133.7	133.24	133.2	133.16	
MTIC332, IV	M1			109.74			
	M2			122.82			
	M3		126.96	126.82	126.92	126.96	
	M4	128.26			128.30	128.34	128.39
	M5		129.19			129.12	129.16
	M6			136.16			
	M7	137.66			137.65	137.64	137.63
B14B03, V	M1			141.99			
	M2			148.29			
	M3		152.32	152.75	152.93	152.69	152.84
	M4	164.8			164.61	164.62	164.6
	M5			166.56			
	M6	172.26	172.28		172.32	172.34	172.34
MTIC48, V	M1			151.77			
	M2			160.09			
	M3	163.99	164.08	163.98	(1)	164.14	163.91
	M4			167.94			
	M5		174.64		174.74	174.64	
MTIC153, VI	M1		144.27			144.34	
	M2	150.82			150.79	150.68	
	M3			152.78			
	M4	159.11					159.02
	M5			165.74			
	M6		171.66	171.63	171.56		171.55
	M7			176.51			
MTIC273, VII	M1	232.72			232.75	232.72	
MTIC135, VIII	M1		112.92		112.74		112.67
	M2			116.11		116.1	
	M3	122.75				122.98	122.84
	M4	124.61		124.66	124.64		

(1) Two copies of a null allele must have been transmitted to BSP S29 from PG-F9; alternatively, a DNA loss might have occurred
