# Supplementary material for: Sexual Polyploidization in Medicago sativa L.: Impact on the Phenotype, Gene Transcription, and Genome Methylation
Source: G3 (Bethesda). 2016 Feb 5;6(4):925–38. doi: 10.1534/g3.115.026021 (PMC4825662; doi:10.1534/g3.115.026021)
Supplement: Supplemental Material [file supp_g3.115.026021_TableS3.pdf]

Table S3. X^2 analysis of segregation mode of the BSP plant S29-4x. No double reduction was assumed

Marker, Chromosome	Origin of alleles	Gamete types	Numbers of progenies			$X^{2\ (1)}$	
			Observed	Expected disomic	Expected tetrasomic	Disomic	Tetrasomic
FMT13, I	PG-F9	M3M3, M3 -	54	59/29.5	49.16	NT/40.69** (2)	2.85
		- -	5	0/29.5	9.83		
MTIC451, II	PG-F9	M4M4, M4 -	38	30	50	4.26*	17.28**
		- -	22	30	10		
MTIC189, III	PG-F9	M2M2, M2 -	45	29	48.33	17.65**	1.37
		- -	13	29	9.66		
MTIC332, IV	PG-F9	M4M7	5	0	9.83	NT	4.50
		M4 -	26	29.5	19.66		
		- M7	18	29.5	19.66		
		- -	10	0	9.83		
B14B03, V	PG-F9	M4M4, M4 -	41	30	50	8.06*	9.73*
		- -	19	30	10		
MTIC48, V	12-P	M5M5, M5 -	51	30	50	29.40**	0.12
		- -	9	30	10		
MTIC153, VI	PG-F9	M2M2, M2 -	54	29.5	49.16	40.69**	2.85
		- -	5	29.5	9.83		
MTIC273, VII	PG-F9	M1M1, M1 -	54	60/30	50	NT/38.40** (2)	1.92
		- -	6	0/30	10		
MTIC135, VIII	12-P	M1M1, M1 -	37	42/21	35	NT/24.38**	0.68
		- -	5	0/21	7		

(1) At the *P*=0.05 probability level, X^2 for 1 df is 3.84; X^2 for 2 df is 5.99; X^2 for 3 df is 7.81; X^2 for 5 df is 11.07. *: significant at $P \leq 0.05$; **: significant at $P \leq 0.01$; NT: non testable because one or more of the expected numbers is 0.
(2) The two figures separated by a slash for expected numbers and Chi square values correspond to different expectations depending on the genotype of a BSP plants for an allele: duplex (former figure) or simplex (latter figure).
