# Supplementary material for: Sexual Polyploidization in Medicago sativa L.: Impact on the Phenotype, Gene Transcription, and Genome Methylation
Source: G3 (Bethesda). 2016 Feb 5;6(4):925–38. doi: 10.1534/g3.115.026021 (PMC4825662; doi:10.1534/g3.115.026021)
Supplement: Supplemental Material [file supp_g3.115.026021_TableS4.pdf]

Table S4. X^2 analysis of segregation mode of the BSP plant S48-4x. No double reduction was assumed

Marker, Chromosome	Origin of alleles	Gamete types	Numbers of progenies			X^{2} [(1)]	
			Observed	Expected disomic	Expected tetrasomic	Disomic	Tetrasomic
FMT13, I	PG-F9	M3M3, M3 -	45	59/29.5	49.16	NT/16.28** (2)	2.11
		- -	14	0/29.5	9.83		
MTIC451, II	PG-F9	M1M4	6	0	10	NT	4.8
		M1 -	16	30	20		
		- M4	24	30	20		
		- -	14	0	10		
MTIC189, III	PG-F9	M4M4, M4 -	48	29	48.33	24.89**	0.01
		- -	10	29	9.66		
MTIC332, IV	12P (M5) PGF9 (M4,M7)	M4M7	6	0	9.5	NT	10.47
		M4 M5	5	14,25	9.5		
		M7M5	11	14,25	9.5		
		M4-	11	14,25	9.5		
		M5-	17	0	9.5		
		M7-	7	14,25	9.5		
B14B03, V	PG-F9	M4M4, M4 -	35	30	50	1.66	27.00**
		- -	25	30	10		
MTIC48, V	12P	M5M5, M5 -	27	29	48.33	0.27	56.40**
		- -	31	29	9.66		
MTIC153, VI	12P (M1) PG-F9 (M2)	M1M1	5	0	9.83	NT	0.48
		M1M2	43	59	39.33		
		M2M2	11	0	9.83		
MTIC273, VII	PG-F9	M1M1, M1 -	33	58/29	48.33	NT/1.10 (2)	29.18**
		- -	25	0/29	9.66		
MTIC135, VIII	PG-F9	M3M3, M3 -	29	21.5	35.83	5.23	7.81
		- -	14	21.5	7.16		

[(1)] At the P=0.05 probability level, X^2 for 1 df is 3.84; X^2 for 2 df is 5.99; X^2 for 3 df is 7.81; X^2 for 5 df is 11.07. *: significant at $P \leq 0.05$; **: significant at $P \leq 0.01$; NT: non testable because one or more of the expected numbers is 0.
(2) The two figures separated by a slash for expected numbers and Chi square values correspond to different expectations depending on the genotype of a BSP plants for an allele: duplex (former figure) or simplex (latter figure).
