# Supplementary material for: Sexual Polyploidization in Medicago sativa L.: Impact on the Phenotype, Gene Transcription, and Genome Methylation
Source: G3 (Bethesda). 2016 Feb 5;6(4):925–38. doi: 10.1534/g3.115.026021 (PMC4825662; doi:10.1534/g3.115.026021)
Supplement: Supplemental Material [file supp_g3.115.026021_TableS5.pdf]

Table S5. X^2 analysis of segregation mode of the BSP plant S60-4x. No double reduction was assumed

Marker, Chromosome	Origin of alleles	Gamete types	Numbers of progenies			X^{2} [(1)]	
			Observed	Expected disomic	Expected tetrasomic	Disomic	Tetrasomic
FMT13, I	PG-F9	M3M3, M3 -	45	60/30	50	NT/15.00** (2)	3.00
		- -	15	0/30	10		
MTIC451, II	PG-F9	M1M1, M1-	50	30	50	26.66**	0.00
		- -	10	30	10		
MTIC189, III	PG-F9	M2M4	13	0	10	NT	5.20
		M2-	22	30	20		
		-M4	12	30	20		
		- -	13	0	20		
MTIC332, IV	12P (M5) PGF9 (M4,M7)	M4M5	17	15	10	NT	35.80**
		M4 M7	10	0	10		
		M5M7	22	15	10		
		M4-	2	15	10		
		M5-	9	0	10		
		M7-	0	15	10		
B14B03, V	PG-F9	M4M4, M4 -	26	29.5	49.16	0.83	65.49**
		- -	33	29.5	9.83		
MTIC153, VI	PG-F9	M4M4, M4 -	48	2 9.5	49.16	23.20**	0.16
		- -	11	29.5	9.83		
MTIC135, VIII	12P (M1) PG-F9 (M3)	M1M1	3	0	4.5	NT	NT/1.50 (2)
		M1M3	21	13.5/27	9/18		
		M3M3	3	0	0/4.5		
		M1 -	0	13.5/-	9/-		
		M3 -	0	0/-	4.5/-		

(1) At the P=0.05 probability level, X^2 for 1 df is 3.84; X^2 for 2 df is 5.99; X^2 for 3 df is 7.81; X^2 for 5 df is 11.07. *: significant at $P \leq 0.05$; **: significant at $P \leq 0.01$; NT: non testable because one ore more of the expected numbers is 0.
(2) The two figures separated by a slash for expected numbers and Chi square values correspond to different expectations depending on the genotype of a BSP plants for an allele: duplex (former figure) or simplex (latter figure).
