# Supplementary material for: Sexual Polyploidization in Medicago sativa L.: Impact on the Phenotype, Gene Transcription, and Genome Methylation
Source: G3 (Bethesda). 2016 Feb 5;6(4):925–38. doi: 10.1534/g3.115.026021 (PMC4825662; doi:10.1534/g3.115.026021)
Supplement: Supplemental Material [file supp_g3.115.026021_TableS6.pdf]

Table S6. **Number of alleles in parents and 4x BSP hybrids. The shaded numbers indicate the presence of less than the maximum possible number of alleles.**

**Marker**	**Chromosome**	**Number of alleles (PG-F9 + 12-P)**	**S29**	**S48**	**S60**
FMT13	I	3	3	3	3
MTIC451	II	4	3	3	3
MTIC189	III	4	3	3	3
MTIC332	IV	4	3	4	3
B14B03	V	3	3	3	3
MTIC48	V	2	1	2	1
MTIC153	VI	4	2	2	2
MTIC273	VII	1	1	1	0
MTIC135	VIII	3	2	2	2
