# Supplementary material for: Sexual Polyploidization in Medicago sativa L.: Impact on the Phenotype, Gene Transcription, and Genome Methylation
Source: G3 (Bethesda). 2016 Feb 5;6(4):925–38. doi: 10.1534/g3.115.026021 (PMC4825662; doi:10.1534/g3.115.026021)
Supplement: Supplemental Material [file supp_g3.115.026021_TableS7.pdf]

Table S7. Leaf morphology traits of 2x and 4x hybrids and their parents. Means followed by different letters are significantly different at *P*<0.05

Plant	Ploidy	[2] Cell surface area, μ^2	Leaf surface area, mm^2	Leaflet lenght/width ratio	Stomata surface, μ^2		Number of stomata per mm^2	
					Upper epidermis	Lower epidermis	Upper epidermis	Lower epidermis
**PARENTS**								
PG-F9	2x	810	172	1.48	171	191	440	334
12P		801	232	2.39	182	189	400	269
**Parental mean**		**805 B**	**195 C**	**1.82 A**	**177 C**	**190 C**	**420 A**	**298 A**
S8	2x	542	282	1.74	215	235	359	337
S16		860	274	1.58	221	253	346	300
S24		848	235	1.72	217	217	342	190
**2x hybrids mean**		**750 B**	**263 B**	**1.69 A**	**218 B**	**233 B**	**348 B**	**264 A**
S29	4x	1131	360	1.13	298	276	215	223
S48		999	323	1.33	305	341	215	199
S60		909	384	1.04	276	284	220	193
**4x hybrids mean**		**1011 A**	**356 A**	**1.17 B**	**292 A**	**301 A**	**217 C**	**205 B**

[2] 19-35 individual cells from at least four different young, fully expanded leaves were measured per plant; for standardization, only cells adjacent to stomata were measured.
