# Supplementary material for: Sexual Polyploidization in Medicago sativa L.: Impact on the Phenotype, Gene Transcription, and Genome Methylation
Source: G3 (Bethesda). 2016 Feb 5;6(4):925–38. doi: 10.1534/g3.115.026021 (PMC4825662; doi:10.1534/g3.115.026021)
Supplement: Supplemental Material [file supp_g3.115.026021_TableS8.pdf]

Table S8. **Fresh biomass yield of 2x and 4x hybrids and their parents. Means followed by different letters are significantly different at *P*<0.05**.

**Plant**	**Ploidy**	**[(1)] Green matter**		
**PARENTS**		**1st cut**	**2nd cut**	**3rd cut**
PG-F9	2x	64.60	42.74	28.91
12P		51.32	47.73	31.93
**Parental mean**		**55.11**	**46.23 B**	**31.03**
**HYBRIDS**				
S8	2x	52.48	47.94	24.74
S16		96.80	47.39	34.43
S24		62.98	73.57	50.50
**2x hybrids mean**		**70.75**	**58.48 B**	**37.90**
S29	4x	70.00	77.34	48.59
S48		73.08	74.33	43.88
S60		63.76	75.35	32.88
**4x hybrids mean**		**68.95**	**75.74 A**	**43.56**

[(1)] Average of 2-6 rooted cuttings per genotype.
