# Supplementary material for: Sexual Polyploidization in Medicago sativa L.: Impact on the Phenotype, Gene Transcription, and Genome Methylation
Source: G3 (Bethesda). 2016 Feb 5;6(4):925–38. doi: 10.1534/g3.115.026021 (PMC4825662; doi:10.1534/g3.115.026021)
Supplement: Supplemental Material [file supp_g3.115.026021_FigureS7.pdf]

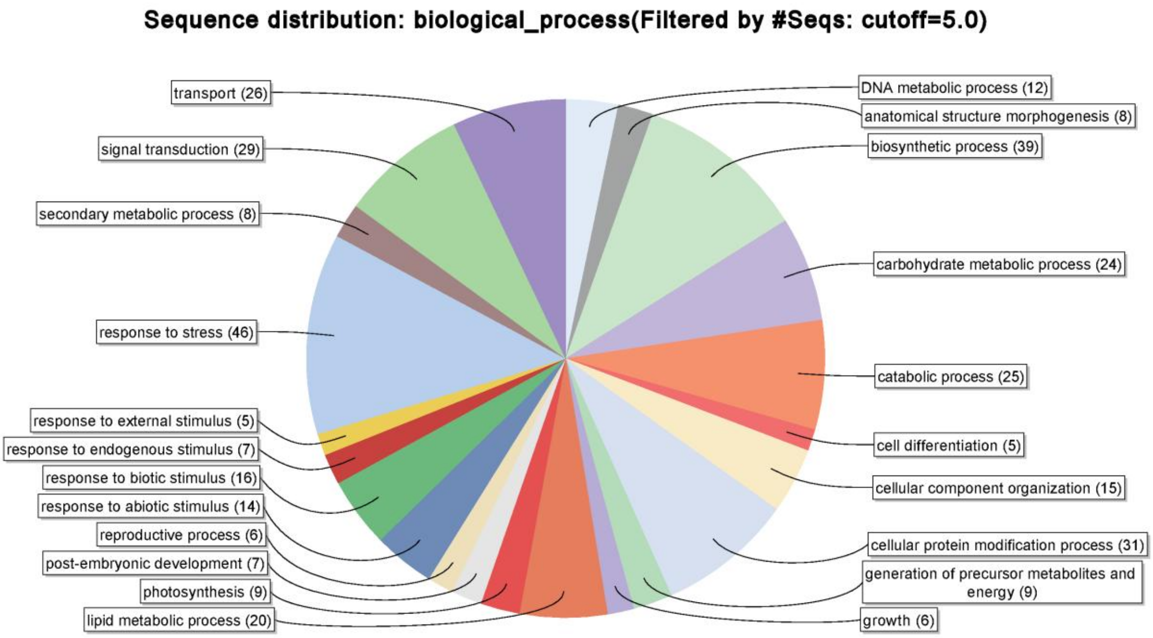

Figure S7. Distribution of GO terms for the Biological process vocabulary among the 341 Ploidy-sensitive genes, adopting the Goslim option for plants.
