# Supplementary material for: Sexual Polyploidization in Medicago sativa L.: Impact on the Phenotype, Gene Transcription, and Genome Methylation
Source: G3 (Bethesda). 2016 Feb 5;6(4):925–38. doi: 10.1534/g3.115.026021 (PMC4825662; doi:10.1534/g3.115.026021)
Supplement: Supplemental Material [file supp_g3.115.026021_FigureS10.pdf]

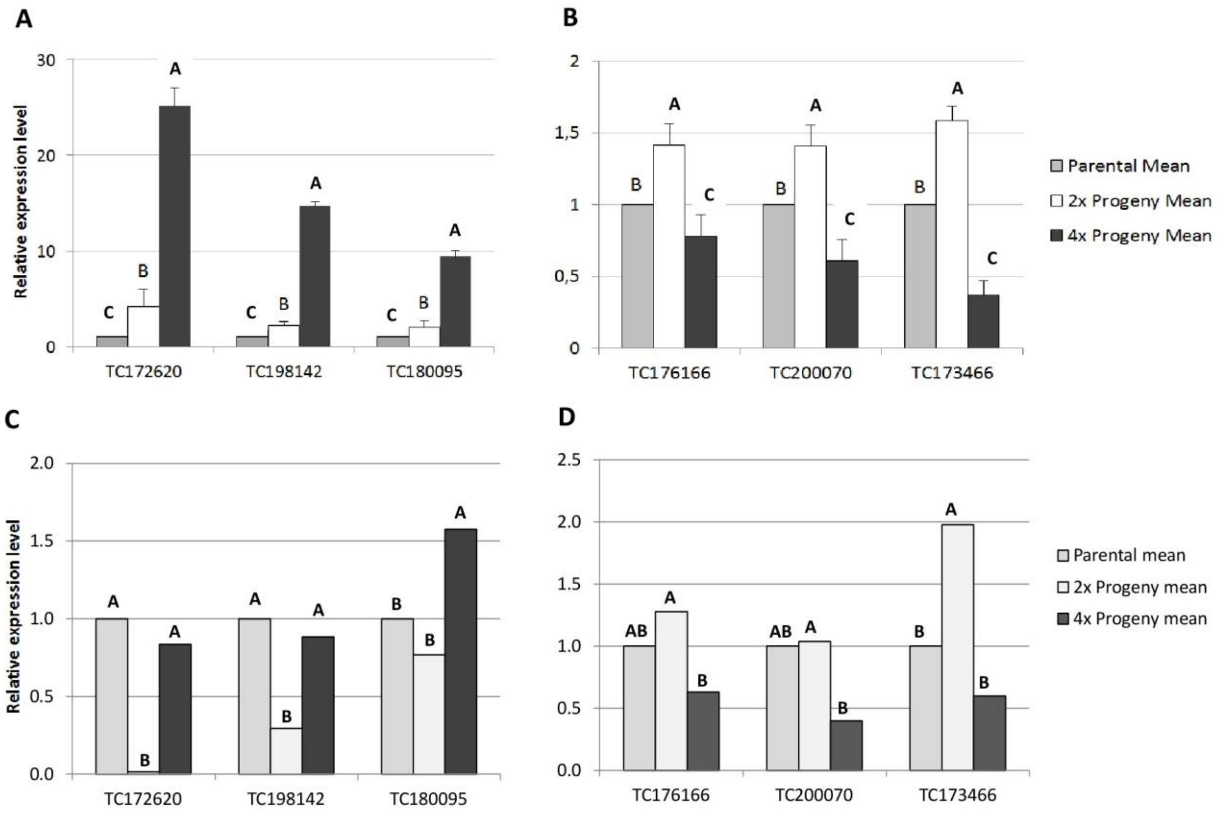

Figure S10. **Comparison of transcriptional levels of six genes among those evidenced by Bingo analysis, as measured by qRT-PCR (A, B) and by microarrays (C, D). Transcript levels are expressed relative to midparent values. A, C: three genes from the 4x>2x group of Table 4; B, D: three genes from the 4x<2x group of Table 4. qRT-PCR data are the averages of 3 biological reps as determined by the $2^{-\Delta\Delta Ct}$ method. Different letters above columns indicate significant differences according to the LSD test (*P*<0.05).**
