# Supplementary material for: Sexual Polyploidization in Medicago sativa L.: Impact on the Phenotype, Gene Transcription, and Genome Methylation
Source: G3 (Bethesda). 2016 Feb 5;6(4):925–38. doi: 10.1534/g3.115.026021 (PMC4825662; doi:10.1534/g3.115.026021)
Supplement: Supplemental Material [file supp_g3.115.026021_FigureS11.pdf]

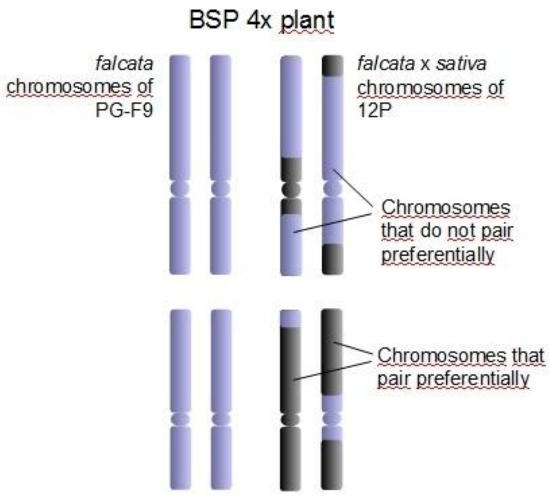

Figure S11. **Model of possible structures of a the four homologous chromosomes in two 4x BSP plants from the cross PG-F9 x 12P. In one plant (above), the four homologs share large portions of subsp. *falcata* sequence (blue), whereas in the other plant (below) the chromosomes from 12P have prevalently *sativa* sequence (grey), and share little sequence with the *falcata* chromosomes from PG-F9.**
